# Development and validation of exhaled breath condensate microRNAs to identify and endotype asthma in children

**DOI:** 10.1371/journal.pone.0224983

**Published:** 2019-11-08

**Authors:** Francisca Castro Mendes, Inês Paciência, António Carlos Ferreira, Carla Martins, João Cavaleiro Rufo, Diana Silva, Pedro Cunha, Mariana Farraia, Pedro Moreira, Luís Delgado, Miguel Luz Soares, André Moreira

**Affiliations:** 1 Serviço de Imunologia Básica e Clínica, Departamento de Patologia, Faculdade de Medicina da Universidade do Porto, Porto, Portugal; 2 EPIUnit—Instituto de Saúde Pública, Universidade do Porto, Porto, Portugal; 3 Institute of Science and Innovation in Mechanical Engineering and Industrial Management (INEGI), Porto, Portugal; 4 Laboratório de Apoio à Investigação em Medicina Molecular (LAIMM), Faculdade de Medicina da Universidade do Porto, Porto, Portugal; 5 Departamento de Biomedicina-Unidade de Biologia Experimental, Centro de Investigação Médica (CIM), Faculdade de Medicina da Universidade do Porto, Porto, Portugal; 6 Pain Group, Instituto de Biologia Molecular e Celular (IBMC), Porto, Portugal; 7 Instituto de Investigação e Inovação em Saúde, Universidade do Porto, Porto, Portugal; 8 Serviço de Imunoalergologia, Centro Hospitalar São João, Porto, Portugal; 9 Faculdade de Ciências da Nutrição e Alimentação da Universidade do Porto, Porto, Portugal; National and Kapodistrian University of Athens, GREECE

## Abstract

Detection and quantification of microRNAs (miRNAs) in exhaled breath condensate (EBC) has been poorly explored. Therefore we aimed to assess miRNAs in EBC as potential biomarkers to diagnose and endotype asthma in school aged children. In a cross sectional, nested case control study, all the asthmatic children (n = 71) and a random sample of controls (n = 115), aged 7 to 12 years, attending 71 classrooms from 20 local schools were selected and arbitrarily allocated to the development or validation set. Participants underwent skin-prick testing, spirometry with bronchodilation, had exhaled level of nitric oxide determined and EBC collected. Based on previous studies eleven miRNAs were chosen and analyzed in EBC by reverse transcription-quantitative real-time PCR. Principal component analysis was applied to identify miRNAs profiles and associations were estimated using regression models. In the development set (n = 89) two clusters of miRNAs were identified. After adjustments, cluster 1 and three of its clustered miRNAs, miR-126-3p, miR-133a-3p and miR-145-5p were positively associated with asthma. Moreover miR-21-5p was negatively associated with symptomatic asthma and positively associated with positive bronchodilation without symptoms. An association was also found between miR-126-3p, cluster 2 and one of its clustered miRNA, miR-146-5p, with higher FEF25-75 reversibility. These findings were confirmed in the validation set (n = 97) where two identical clusters of miRNAs were identified. Additional significant associations were observed between miR-155-5p with symptomatic asthma, negative bronchodilation with symptoms and positive bronchodilation without symptoms. We showed that microRNAs can be measured in EBC of children and may be used as potential biomarkers of asthma, assisting asthma endotype establishment.

## Introduction

Pediatric asthma is a highly-prevalent heterogeneous disease characterized by variable airflow obstruction with cough, dyspnea and wheezing. Frequently, asthma is described in terms of disease phenotypes, since phenotypic characteristics may be easily recognizable. However, underlying biology is complex. In this context, the term endotype was created to cluster asthmatics not only by their phenotypical characteristics but also by the pathophysiological features of the disease [1] and may explain the clinical presentation, epidemiology, and response to different treatments.

Most of asthma biomarkers used for clinical purposes are sampled in blood, exhaled breath, or urine. Induced sputum remains available only in specialized centers. Collection of exhaled breath condensate (EBC) is simple, safe, non-invasive and highly repeatable, and potentially allows the assessment of biomarkers of airway inflammation, being increasingly used as a tool for biomarker discovery [2]. In the EBC matrix a wide number of inflammatory mediators can be measured and several studies have focused on non-volatile organic compounds, like eicosanoids [3], 8-isoprostane [4], and more recently microRNAs (miRNAs) [5–7] due to their known relationship from diagnosis to asthma control. Accordingly, diagnostic biomarkers are needed to appropriately endotype children with asthma and enable the selection of a more personalized therapy. Furthermore, it is essential to improve biomarkers diagnostic accuracy to introduce them into clinical practice.

MicroRNAs are highly conserved small noncoding single-stranded RNAs of 18–25 nucleotides that act as regulators of gene expression, modulate almost all biological processes and are essential for maintaining cellular homeostasis [8]. Specific miRNAs have been found to have critical roles in regulating key pathogenic mechanisms in allergic inflammation including polarization of adaptive immune responses and activation of T cells, through miR-21, which promotes Th2 responses by targeting IL-12 expression, and miR-146. Additionally, it includes the regulation of eosinophil development by miR-21 and miR-223 and modulates IL-13-driven epithelial responses. However, research focused on miRNAs as potential biomarkers to diagnose or endotype asthma is scarce [5, 9, 10].

Finding miRNAs, as an individual or as a group, in lung airways of children may allow the possibility of their use as biomarkers, constituting a powerful tool of diagnostic and prognostic value in asthma. Furthermore, many endotypes have been proposed however, they have not yet been widely accepted. As such, we aimed to assess miRNA in exhaled breath condensate as potential biomarkers to diagnose and endotype asthma of school-aged children, both in a development and in a validation set.

## Methods

### Participants and study design

**Fig 1:**
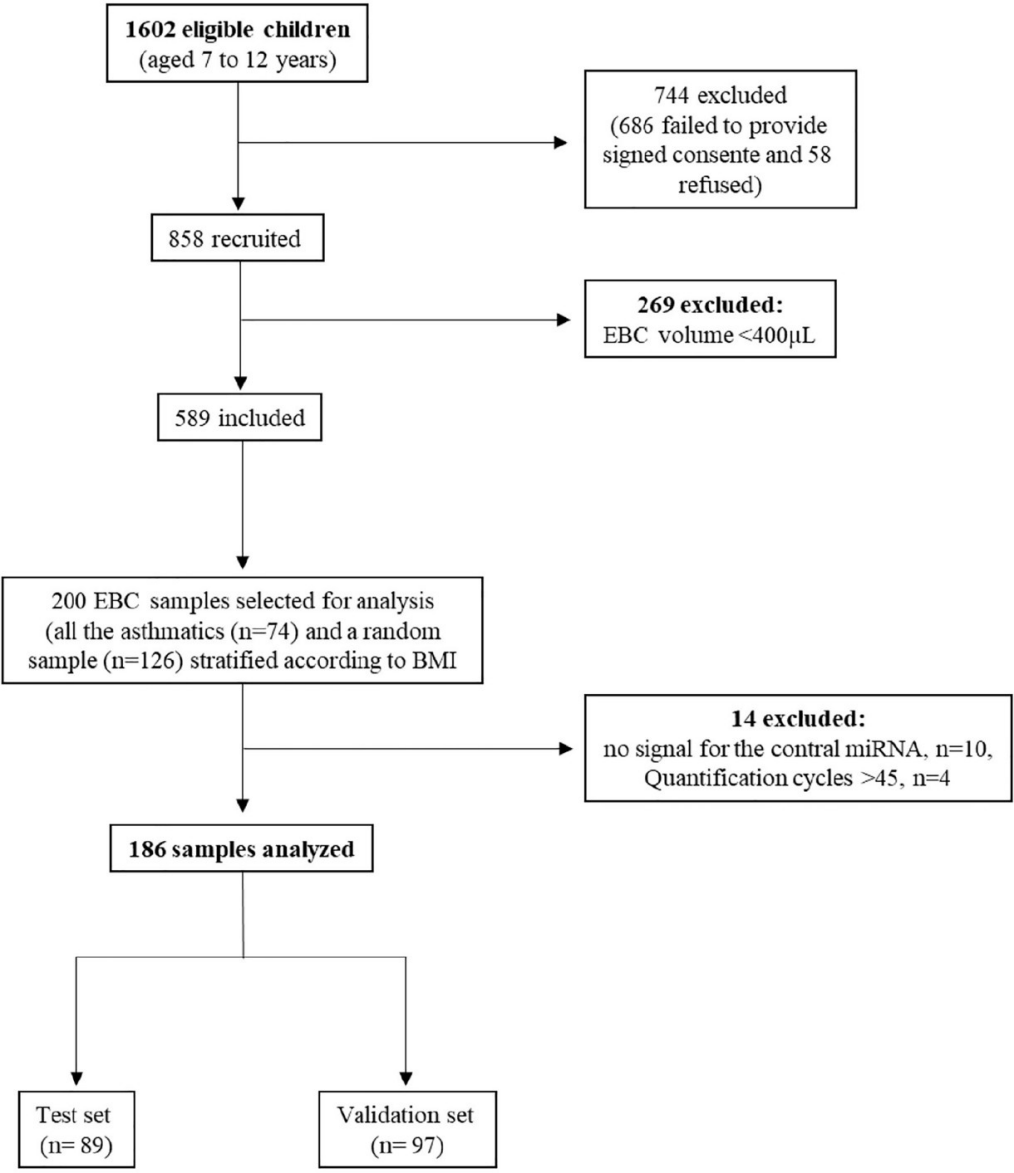
Flow chart of the participants included for exhaled breath condensate (EBC) miRNAs analysis.

Study population was drawn from a cross sectional survey conducted between January 2014 and March 2015, that enrolled 1602 children aged 7 to 12 years old attending the 3^rd^ and 4^th^ grades from 20 primary schools in Porto, Portugal [11]. The participation rate of the study was 57% (n = 916). Those who refused to perform the clinical tests were excluded (n = 58). We assessed symptoms, atopic status, airway inflammation and measured lung function (n = 858). Children not being able to comply with study procedures or who failed to provide an exhaled breath condensate volume of at least 400 μL were excluded (n = 269). Then, all the children with asthma (n = 71) and a random sample of the remaining children (n = 115), stratified for body mass index categories, based on a case: control ratio of 1:2, were selected for this nested case-control study, which analyzed the exhaled breath condensate miRNA profile. Considering a confidence level of 95% and the observed asthma prevalence of 38.2%, the estimated confidence interval for the sample size was ±5.95. The 186 included participants were randomly allocated (1:1) to the development or validation set (Fig 1).

Asthma was defined using a gold standard definition based on a positive bronchodilation (BD) defined as at least 12% and over 200mL increase in FEV1 after inhaling 400 μg of salbutamol; or had self-reported medical diagnosis of asthma with symptoms (wheezing, dyspnea or dry cough) in the previous year [12]. Additionally, different asthma phenotypes were created based on classical definitions that involved several asthma features and traits [13]: i) allergic asthma, defined by positive skin prick tests (SPT); ii) eosinophilic asthma, defined by exhaled nitric oxide above 35 ppb; iii) obese asthma, defined by overweight or obesity; iv) persistent asthma, defined by current use of anti-asthma medication; v) symptomatic asthma, defined by current symptoms; vi) positive bronchodilation with asthma symptoms (BD+S+); vii) positive bronchodilation without asthma symptoms (BD+S-); and viii) negative bronchodilation with asthma symptoms (BD-S+). Characteristics of participants from development and validation set were not significantly different (Table 1). Moreover, phenotypic characteristics of children with asthma are presented in Table 2.

**Table 1:** Characterization of the participants.

	*Development set*, *n = 89*	*Validation set*, *n = 97*	*Total*, *n = 186*	*p-value*
**Age (years)**	8.73 ±0.84	8.70±0.84	8.71 ±0.84	0.89
**Female, n (%)**	43 (48.31)	52 (53.61)	95 (51.08)	0.47
**Atopic, n (%)^a^**	32 (35.96)	40 (41.23)	72 (38.71)	0.46
**BMI categories, n (%)^b^**				0.24
Underweight	8 (9.00)	3 (3.09)	11 (5.90)	
Normal weight	38 (42.70)	36 (37.11)	74 (39.78)	
Overweight	22 (24.72)	30 (30.93)	52 (27.96)	
Obese	21 (23.60)	28 (28.87)	49 (26.34)	
**Current symptoms^c^**				
Breathing difficulties, n (%)	19 (21.34)	**16 (16.49)**	35 (18.82)	0.47
Irritative cough, n (%)	34 (38.20)	**33 (34.02)**	67 (36.02)	0.48
**Lung function**				
FVC, L	1.96 (1.72; 2.12)	1.94 (1.70; 2.12)	1.96 (1.71; 2.12)	0.99
FVC, % predicted	107.97 (96.93; 116.09)	105.91 (99.00; 118.51)	107.86 (98.26; 117.91)	0.82
FEV1, L	1.82 (1.58; 1.97)	1.77 (1.60; 1.95)	1.80 (1.59; 1.96)	0.76
FEV1, % predicted	102.99 (92.30; 111.83)	103.33 (95.65; 112.53)	103.16 (93.92; 111.98)	0.66
FEF25-75, L/s	2.30 (1.88; 2.73)	2.27 (1.76; 2.76)	2.29 (1.84; 2.75)	0.35
FEF25-75, % predicted	99.29 (82.93; 108.93)	96.73 (80.46; 125.61)	97.90 (81.50; 115.86)	0.58
FEV1 reversibility, %	4.57 (1.46; 11.5)	6.67 (2.29; 12.3)	5.52 (1.87; 12.2)	0.66
FEF25-75 reversibility, %	14.4 (2.59; 23.3)	12.4 (3.57; 22.9)	13.7 (3.17; 23.2)	0.97
**Asthma^d^, n (%)**	40 (44.94)	31 (31.96)	71 (38.17)	0.07
Allergic asthma	18 (20.22)	14 (14.43)	32 (17.20)	0.98
Eosinophilic asthma	12 (13.48)	7 (7.22)	19 (10.22)	0.48
Obese asthma	15 (16.85)	11 (11.34)	26 (13.98)	0.86
Persistent asthma	25 (28.09)	15 (15.46)	55 (29.57)	0.23
Symptomatic asthma	26 (29.21)	17 (17.53)	43 (23.12)	0.39
BD+S+	7 (7.87)	3 (3.09)	10 (5.38)	0.35
BD+S-	14 (15.73)	14 (14.43)	28 (15.05)	0.39
BD-S+	19 (21.35)	14 (14.43)	33 (17.74)	0.85
**Exhaled NO, ppb**	11.00 (7.00; 20.00)	13.00 (6.00; 23.00)	12.00 (6.00; 21.00)	0.57
**MicroRNA**				
miR-21-5p	3.22 (1.46; 5.99)	2.17 (0.59; 4.56)	2.51 (0.97; 5.50)	0.09
miR-126-3p	0.05 (1.37x10^-4^; 0.28)	0.06 (1.37x10^-4^; 0.35)	0.05 (1.37x10^-4^; 0.29)	0.73
miR-133a-3p	0.02 (6.9x10^-5^; 0.31)	0.02 (6.9x10^-5^; 0.42)	0.02 (6.85x10^-5^; 0.35)	0.41
miR-145-5p	0.50 (0.25; 1.44)	0.59 (0.20; 1.07)	0.55 (0.22; 1.25)	0.60
miR-146a-5p	6.65x10^-4^ (6.65x10^-4^; 0.03)	6.65x10-4 (6.65x10^-4^; 0.03)	6.65x10^-4^ (6.65x10^-5^; 0.03)	0.69
miR-155-5p	1.19x10^-4^ (1.19x10^-4^; 0.02)	1.19x10^-4^ (1.19x10^-4^; 1.19x10^-4^)	1.19x10^-4^ (1.19x10^-4^; 2.37x10^-4^)	0.06
miR-221-3p	0.09 (1.89x10^-3^; 0.30)	0.06 (1.89x10^-3^; 0.28)	0.08 (1.89x10^-3^; 0.29)	0.76
miR-328-3p	0.56 (0.13; 1.20)	0.45 (0.12; 1.04)	0.48 (0.12; 1.16)	0.45
miR-423-3p	1.07x10^-3^(1.07x10^-3^; 0.09)	1.07x10^-3^(1.07x10^-3^; 0.01)	1.07x10^-3^ (1.07x10^-3^; 0.07)	0.14

Data are expressed as medians (25^th^-75^th^ percentile) unless otherwise stated.

^a^: atopy defined by a positive skin prick test to at least one of the allergens

^b^: Body Mass Index according to US Centers for Disease Control

^c^: symptoms in the previous 3 months

^d^: asthma was defined based on positive bronchodilation or self-reported medical diagnosis with reported symptoms in the previous year

SD: standard deviation

FVC: forced vital capacity

FEV1: forced expiratory volume in the first second

FEF25-75: forced expiratory flow middle portion of FVC

FEV1 reversibility: forced expiratory volume in the first second after bronchodilation

FEF25-75 reversibility: forced expiratory flow middle portion of FVC after bronchodilation

**Table 2:** MiRNA levels according to asthma phenotypes.

**Development set**									
	***Asthma***^***a***^ ***(n = 40)***	***Allergic asthma (n = 18)***	***Eosinophilic asthma (n = 12)***	***Obese asthma (n = 15)***	***Persistent asthma (n = 25)***	***Symptomatic asthma (n = 26)***	***BD+S+ (n = 7)***	***BD+S- (n = 14)***	***BD-S+ (n = 19)***
**MicroRNA**									
miR-21-5p	1.83 (0.52; 4.20)	1.61 (0.22; 4.34)	1.80 (0.56; 3.80)	1.36 (0.17; 4.06)	1.75 (0.40; 4.34)	1.17 (0.40; 3.61)	1.36 (0.55; 2.71)	2.85 (1.57; 5.62)	0.85 (0.22; 4.34)
miR-126-3p	0.11 (1.37x10^-4^; 0.96)	2.79x10^-3^ (1.37x10^-4^; 0.37)	7.85x10^-3^ (1.40x10^-4^; 0.24)	0.20 (1.37x10^-4^; 1.42)	0.06 (1.37x10^-4^; 1.42)	0.04 (1.37x10^-4^; 0.20)	1.37x10^-4^ (1.37x10^-4^; 0.27)	0.44 (1.37x10^-4^; 1.34)	0.09 (1.37x10^-4^; 0.20)
miR-133a-3p	0.08 (6.90x10^-5^; 0.53)	0.01 (6.85x10^-5^; 0.42)	0.02 (7.00x10^-5^; 0.38)	0.11 (6.85x10^-5^; 0.44)	0.02 (6.85x10^-5^; 0.44)	2.24x10^-3^ (6.85x10^-5^; 0.33)	6.85x10^-5^ (6.85x10^-5^; 0.32)	0.26 (0.04; 1.24)	2.96x10^-3^ (6.85x10^-5^; 0.44)
miR-145-5p	0.59 (0.23; 1.32)	0.54 (0.15; 1.30)	0.36 (0.11; 0.84)	0.59 (0.24; 1.30)	0.59 (0.20; 1.35)	0.43 (0.15; 1.25)	0.36 (1.20x10^-3^; 0.63)	0.76 (0.49; 2.56)	0.59 (0.20; 1.35)
miR-146a-5p	6.65x10^-4^ (6.65x10^-4^; 0.04)	6.65x10^-4^ (6.65x10^-4^; 0.04)	6.67x10^-4^ (6.67x10^-4^; 0.05)	6.65x10^-4^ (6.65x10^-4^; 0.04)	4.08x10^-3^ (6.65x10^-4^; 0.05)	6.67x10^-4^ (6.67x10^-4^; 0.05)	6.67x10^-4^ (6.67x10^-4^; 0.05)	6.67x10^-4^ (6.67x10^-4^; 0.04)	6.65x10^-4^ (6.65x10^-4^; 0.03)
miR-155-5p	1.19x10^-4^ (1.19x10^-4^; 1.19x10^-4^)	1.19x10^-4^ (1.19x10^-4^; 1.19x10^-4^)	1.19x10^-4^ (1.19x10^-4^; 1.19x10^-4^)	1.19x10^-4^ (1.19x10^-4^; 1.19x10^-4^)	1.19x10^-4^ (1.19x10^-4^; 1.19x10^-4^)	1.19x10^-4^ (1.19x10^-4^; 1.19x10^-4^)	1.19x10^-4^ (1.19x10^-4^; 1.19x10^-4^)	1.19x10^-4^ (1.19x10^-4^; 1.19x10^-4^)	1.19x10^-4^ (1.19x10^-4^; 1.19x10^-4^)
miR-221-3p	0.04 (1.89x10^-3^; 0.29)	0.03 (1.89x10^-3^; 0.13)	0.06 (1.89x10^-3^; 0.46)	0.01 (1.89x10^-3^; 0.33)	0.05 (1.89x10^-3^; 0.24)	0.02 (1.89x10^-3^; 0.21)	1.89x10^-3^ (1.89x10^-3^; 0.36)	0.15^3^ (1.89x10^-3^; 0.83)	0.03 (1.89x10^-3^; 0.10)
miR-328-3p	0.44 (0.05; 1.08)	0.44 (0.08; 0.91)	0.42 (0.03; 0.90)	0.24 (5.98x10^-3^; 1.00)	0.33 (0.02; 1.00)	0.29 (5.98x10^-3^; 0.90)	0.24 (5.98x10^-3^; 0.68)	0.85 (0.27; 1.78)	0.33 (5.98x10^-3^; 1.48)
miR-423-3p	1.07x10^-3^ (1.07x10^-3^; 1.07x10^-3^)	1.07x10^-3^ (1.07x10^-3^; 2.15x10^-3^)	1.07x10^-3^ (1.07x10^-3^; 0.04)	1.07x10^-3^ (1.07x10^-3^; 1.07x10^-3^)	1.07x10^-3^ (1.07x10^-3^; 1.07x10^-3^)	1.07x10^-3^ (1.07x10^-3^; 1.07x10^-3^)	1.07x10^-3^ (1.07x10^-3^; 1.07x10^-3^)	1.07x10^-3^ (1.07x10^-3^; 1.07x10^-3^)	1.07x10^-3^ (1.07x10^-3^; 2.15x10^-3^)
**Validation set**									
	***Asthma***^***a***^ ***(n = 31)***	***Allergic asthma (n = 14)***	***Eosinophilic asthma (n = 7)***	***Obese asthma (n = 11)***	***Persistent asthma (n = 15)***	***Symptomatic asthma (n = 17)***	***BD+S+ (n = 3)***	***BD+S- (n = 14)***	***BD-S+ (n = 14)***
miR-21-5p	3.37 (1.35; 5.93)	3.29 (0.67; 3.91)	3.22 (1.35; 3.91)	2.88 (1.54; 3.91)	3.44 (2.00; 4.04)	3.22 (2.00; 3.81)	2.88 (0.25; 10.9)	3.29 (2.00; 3.81)	3.29 (2.00; 3.81)
miR-126-3p	0.03 (1.37x10^-4^; 0.29)	0.03 (1.37x10^-4^; 0.24)	7.85x10^-3^ (1.37x10^-4^; 0.24)	0.15 (7.05x10^-3^; 1.18)	0.02 (1.37x10^-4^; 0.15)	0.03 (1.37x10^-4^; 0.15)	0.05 (2.72x10^-4^; 0.29)	0.03 (1.37x10^-4^; 0.15)	0.03 (1.37x10^-4^; 0.15)
miR-133a-3p	0.03 (6.90x10^-5^; 0.38)	0.05 (6.85x10^-5^; 0.38)	0.03 (6.90x10^-5^; 0.43)	0.06 (6.85x10^-5^; 0.37)	0.06 (6.85x10^-5^; 0.38)	0.03 (6.85x10^-5^; 0.12)	8.76x10^-3^ (6.85x10^-5^; 0.07)	0.05 (6.85x10^-5^; 0.38)	0.05 (6.85x10^-5^; 0.38)
miR-145-5p	0.44 (0.17; 1.18)	0.74 (0.15; 1.60)	0.17 (9.23x10-3; 1.01)	0.48 (0.24; 1.18)	0.49 (0.25; 1.60)	0.48 (0.19; 1.04)	0.29 (0.19; 0.54)	0.49 (0.11; 1.33)	0.49 (0.11; 1.33)
miR-146a-5p	6.65x10^-4^ (6.65x10^-4^; 0.02)	6.65x10^-4^ (6.65x10^-4^; 0.02)	6.65x10^-4^ (6.65x10^-4^; 6.65x10^-4^)	6.65x10^-4^ (6.65x10^-4^; 6.65x10^-4^)	6.65x10^-4^ (6.65x10^-4^; 6.65x10^-4^)	6.65x10^-4^ (6.65x10^-4^; 6.65x10^-4^)	0.03 (6.65x10-3; 0.32)	6.65x10^-4^ (6.65x10^-4^; 6.65x10^-4^)	6.65x10^-4^ (6.65x10^-4^; 6.65x10^-4^)
miR-155-5p	1.19x10^-4^ (1.19x10^-4^; 1.19x10^-4^)	1.19x10^-4^ (1.19x10^-4^; 1.19x10^-4^)	1.19x10^-4^ (1.19x10^-4^; 1.19x10^-4^)	1.19x10^-4^ (1.19x10^-4^; 2.07x10^-3^)	1.19x10^-4^ (1.19x10^-4^; 1.60x10^-3^)	1.19x10^-4^ (1.19x10^-4^; 1.19x10^-4^)	1.19x10^-4^ (1.19x10^-4^; 0.03)	1.19x10^-4^ (1.19x10^-4^; 1.19x10^-4^)	1.19x10^-4^ (1.19x10^-4^; 1.19x10^-4^)
miR-221-3p	0.13 (1.89x10^-3^; 0.44)	0.12 (1.89x10^-3^; 0.42)	0.08 (1.89x10^-3^; 0.61)	0.18 (1.89x10^-3^; 0.69)	0.18 (1.89x10^-3^; 0.61)	0.13 (1.89x10^-3^; 0.49)	0.13 (1.89x10^-3^; 0.46)	0.15 (1.89x10^-3^; 0.61)	0.15 (1.89x10^-3^; 0.61)
miR-328-3p	0.55 (5.98x10^-4^; 1.37)	1.04 (0.55; 2.32)**	0.61 (0.03; 1.61)	0.26 (0.08; 0.98)	0.89 (0.42; 2.32)*	0.63 (0.42; 1.37)	0.25 (5.98x10^-4^; 0.63)	0.94 (0.47; 1.61)	0.94 (0.47; 1.61)*
miR-423-3p	1.07x10^-3^ (1.07x10^-3^; 0.10)	1.07x10^-3^ (1.07x10^-3^; 0.05)	1.07x10^-3^ (1.07x10^-3^; 0.09)	1.07x10^-3^ (1.07x10^-3^; 0.19)	1.07x10^-3^ (1.07x10^-3^; 0.10)	1.07x10^-3^ (1.07x10^-3^; 0.10)	0.19 (1.07x10^-3^; 0.22)	1.07x10^-3^ (1.07x10^-3^; 0.07)	1.07x10^-3^ (1.07x10^-3^; 0.07)

Data are expressed as medians (25^th^-75^th^ percentile)

^a^: asthma was defined based on positive bronchodilation or self-reported medical diagnosis with reported symptoms in the previous year

Allergic asthma: defined by “asthma” in a child with positive skin prick test

Eosinophilic asthma: defined by “asthma” in a child with exhaled nitric oxide above 35 ppb

Obese asthma: defined by “asthma” in an overweight or obese child

Persistent asthma: defined by “asthma” in a child currently using anti-asthma medication

Symptomatic asthma: defined by “asthma” in a child with current symptoms

BD+S+: Positive bronchodilation with asthma symptoms defined by “asthma” in a child with a current positive bronchodilation test and symptoms

BD+S-: Positive bronchodilation without asthma symptoms defined by “asthma” in a child with a current positive bronchodilation test and without symptoms

BD-S+: Negative bronchodilation with asthma symptoms defined by “asthma” in a child with a current negative bronchodilation test and with symptoms

Significant differences in bold

*: p-value <0.05

**: p-value <0.01

The study was approved by the Ethical Committee of the University Hospital São João (approval 248–13). Every procedure was in accordance with Helsinki Declaration, and written consent was obtained from children’s legal guardians.

### Questionnaires

A self-administered ISAAC-based questionnaire [14], which included questions regarding social, demographic and behavioral characteristics, as well as questions regarding respiratory/allergic health and current symptoms was filled by children caregivers and reviewed by a research nurse. Wheezing symptoms were defined by a positive answer to the question “Did your child have wheezing or whistling in the chest, in the past 12 months?”. Cough symptoms were defined by a positive answer for any of the two following questions “Did your child suffer from night coughing in the last 12 months?” or “Did your child suffer off recurrent coughing more than three months in the last year?”. Children were considered to have current symptoms in the previous 3 months if there was a positive answer to the question “During the past 3 months, has your child had any of the following symptoms?”: “Irritative cough” and “Breathing difficulties”. The current use of anti-asthma medication was defined by a positive answer to the question “In the past 12 months, has your child used any medicines, pills, puffers or other medication for wheezing or asthma?”

### Assessments

Data on anthropometry (weight and height) were used to calculate body mass index (BMI). Weight was measured using a digital scale (Tanita^™^ BC-418 Segmental Body Analyzer) and height was measured with a portable stadiometer. Body mass index was defined as kg per square meters (kg/m^2^) and classified in different categories (thinness, normal weight, overweight, an obesity) according to age- and sex-specific percentiles. According to the US Centers for Disease Control and Prevention (CDC) the BMI of children was defined overweight and obese when the percentile was between 85^th^ and <95^th^ and ≥ 95^th^, respectively [15].

Lung function and airway reversibility were assessed by spirometry according to the official ATS/ERS guidelines and were recorded before and 15 minutes after the inhalation of 400μg of salbutamol.

To assess airway inflammation levels of exhaled nitric oxide (NO) were measured using the NObreath analyzer (Bedfont Scientific Ltd., Rochester, Kent, UK). The results were expressed as parts per billion (ppb) and stratified according to the official ATS guidelines for children [16]. Eosinophilic inflammation was defined by exhaled nitric oxide levels above 35 ppb.

Skin-prick-tests (SPT) were performed on children forearm to evaluate allergic sensitization using a QuickTest^TM^ (Panatrex Inc., Placentia, California, USA applicator) which contained house dust mite, weed pollen mix (*Urtica dioica*, *Plantago lanceolate* and *Artemisia vulgaris*), grass pollen mix (*Agrostis stolonifera*, *Anthoxathum odoratum*, *Dactylis glomerata*, *Lolium perenne*, *Arrhenatherium elatius*, *Festuca rubra*, *Poa pratensis*, *Holcus lanatus*, *Phleum pratense*, *Secale cereal*), cat dander, dog dander, *Alternaria alternata*, negative control (extracts dilutant), and a positive control consisting of histamine at 10mg/mL (Hall Allergy, Netherlands). After15 minutes, results were read. Atopy was defined based on a positive SPT (wheal ≥3 mm diameter) to at least one of the allergens.

### Exhaled breath condensate collection and miRNA quantification

From included participants, EBC samples were collected, using an exhaled air condensing system (portable Turbo DECCS) [17]. According with manufacturer’s instructions, device was cooled to 0°C before collection.

EBC samples were acquired for at least 15 minutes of normal breathing, wearing a nose clip. In general, samples volume varies between 800 to 1500 μL. Variation in volume is associated with the tidal and minute volumes of the lungs of the children [18]. In this study, EBC samples with volume lower than 400 μL were excluded.

After collection, samples were stored in capped glass tubes at -80°C, until analysis. Procedure was performed under a laminar flow cabinet to ensure a controlled environment and avoid possible contamination by environmental air [19].

Based on previous studies [7, 10, 20–27], eleven specific miRNAs was chosen for analysis: let7a-5p, miR21-5p, miR126-3p, miR133a-3p, miR145-5p, miR146a-5p, miR155-5p, miR221-3p, miR328-3p, miR-1248 and miR-423-3p.

Total RNA was extracted by guanidinium thiocyanate-phenol-chloroform extraction from 0.5 mL of EBC samples using Tri Reagent LS (Sigma-Aldrich). Prior to RNA extraction, the EBC samples were spiked with synthetic UniSp6 RNA from Exiqon (Qiagen USA) for extraction control purposes. The extracted RNA was quality controlled with a Bioanalyzer (Agilent Technologies, USA) using the Small RNA Kit (Agilent) according to manufacturer’s instructions. Reverse transcription (RT) and real-time quantitative (q) PCR were carried out with reagents employing Locked Nucleic Acid (LNA^™^) technology (Exiqon). LNAs are a class of high-affinity RNA analogues in which the ribose ring is “locked” in the ideal conformation for Watson-Crick binding resulting in unprecedented sensitivity and specificity for the detection of small or highly similar RNA targets. RNA extracted from each EBC sample was reverse transcribed in duplicate with the Universal cDNA synthesis kit II (Exiqon). Ten microliters of RNA solution were used per RT reaction. The resulting cDNA was diluted 10x and subjected to quantification by real-time quantitative PCR in 10 μL reactions using miRNA-specific LNA oligos and ExiLENT SYBR Green master mix (Exiqon).

Quantitative PCR was performed using a StepOnePlus Real-Time PCR System (Applied Biosystems) under a 2-step program: 95°C for 10 sec, 60°C for 1 min, with 45 cycles. All assays were done in duplicate; one no-template and one positive control (UniSp6) were used in each experiment. For inter-plate calibration, one specific sample was amplified for UniSp6 in duplicate, in the same well location of each individual plate. miR-1248

GenEX software (MultiD Analyses AB, Göteborg, Sweden) was used for data normalization and analysis. Data were normalized using let7a-5p, as selected by NormFinder and geNorm, after assessing that this miRNA was relatively invariantly expressed across our samples. There are contradictory and inconsistent results implicating let7a in asthma. Whereas a few studies indicate that let7a-5p is deregulated in asthma [28, 29], other studies challenge these results showing no differences in let7a expression between healthy subjects and asthma patients [30, 31]. A common feature to all these studies is the relatively small sample size used for analysis.

### Statistical analyses

Principal component analysis (PCA) was applied to identify miRNAs clusters. As previously described, PCA is a technique of powerful dimensionality reduction, which allows to identify data patterns, compacting them by reducing the number of dimensions [32]. This technique is based on the calculation of the covariance matrix eigenvectors and eigenvalues. The eigenvector with the highest eigenvalue is frequently called first principal component, characterized by its linear combination of original variables that captures the maximum variance in the dataset. Moreover, it is applied without considering the correlation between the dependent and the independent variables [33]. In the present study, kaiser normalization was performed, and adequacy values were above 0.6, value required for a good factor analysis. Only components with eigenvalues above 1.0 were retained in the solution. Factor loadings in the factorial analysis can be interpreted as correlation coefficients between miRNAs and those with a moderate or strong correlation coefficient, above 0.5, were considered as significantly contributing to a cluster. In this analysis, miR-1248 was not included, since it was absent in over 80% of the samples. This technique examined the correlational structure of the data of the remaining 9 miRNAs.

The normality distribution of continuous variables was tested by the Kolmogorov–Smirnov test. Comparisons between clusters were performed using the Mann–Whitney test, when non-Gaussians distributions were observed, and chi-squared test for categorical data.

Logistic and linear regression models were performed to assess the association between clusters and individual miRNAs, as independent variables, and asthma, asthma phenotypes, current symptoms, exhaled nitric oxide and lung function, as dependent variables. Age, sex, exhaled NO, atopy, body mass categories, anti-asthma medication, positive bronchodilation, reported symptoms in the past 12 months and asthma were analyzed as potential confounders. Significant differences were reported with an α-value inferior to 5% (p<0.05).

All statistical tests were performed using the SPSS statistical package software v25.0 (IBM, USA).

## Results

In both development and validation set, principal component analysis exhibited two clusters that satisfactorily described the distributions of microRNAs levels in children. Cluster 1 showed high positive loadings of miR-126-3p, miR-133a-3, miR-145-5p, miR-221-3p and miR-328-3p and cluster 2 of miR-146a-5p and miR-423-3p (S1 Fig). Training set additionally encompassed miR-21-5p in cluster 2.

**Fig 2:**
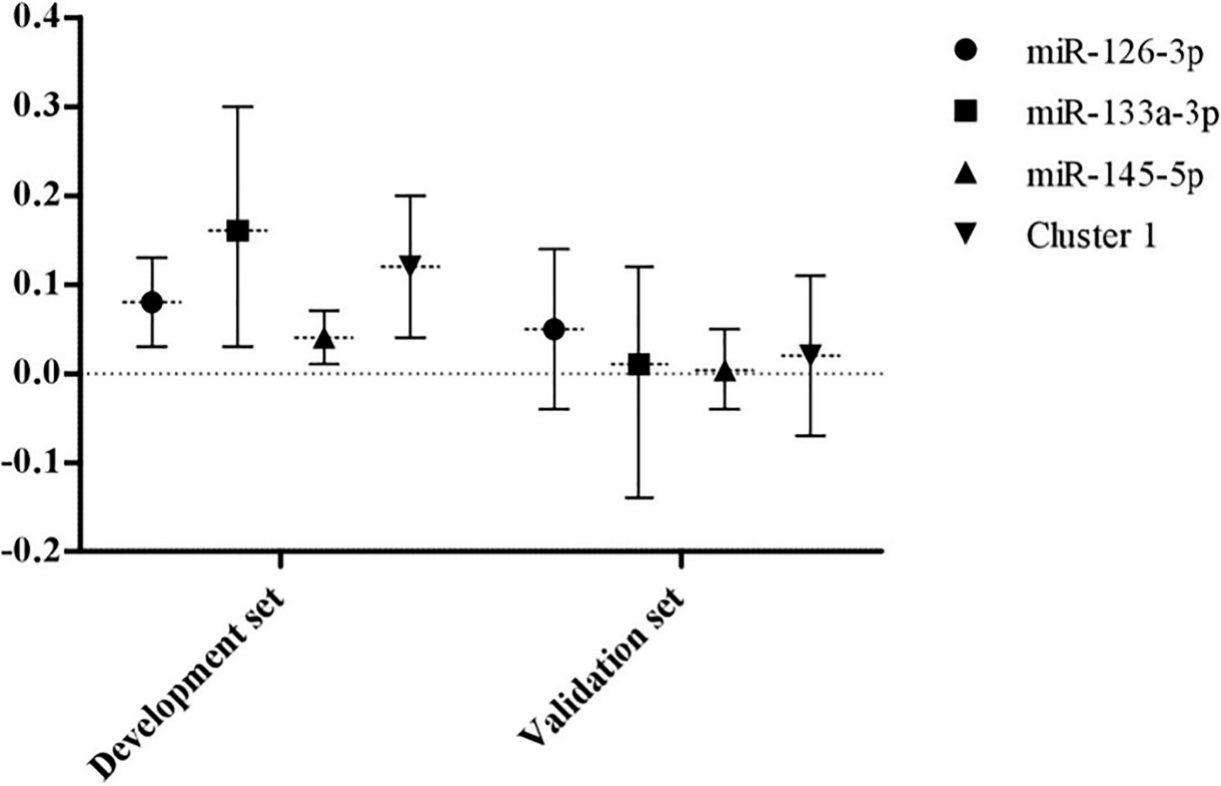
Association between miRNAs and asthma. Associations between cluster 1, miR-126-3p, miR-133a-3p and miR-145-5p with asthma in both development and validation set. Cluster 1: typified by miR-126-3p, miR-133a-3p, miR-145-5p, miR-221-3p and miR-328-3p; asthma: defined based on positive bronchodilation or self-reported medical diagnosis with reported symptoms in the previous year.

After adjustments, cluster 1 and three of its clustered miRNAs, miR-126-3p, miR-133a-3p and miR-145-5p were positively associated with asthma, defined by positive BD or self-reported medical diagnosis of asthma with symptoms in the previous year (Fig 2). These findings were similarly observed in the validation set (Fig 2).

**Fig 3:**
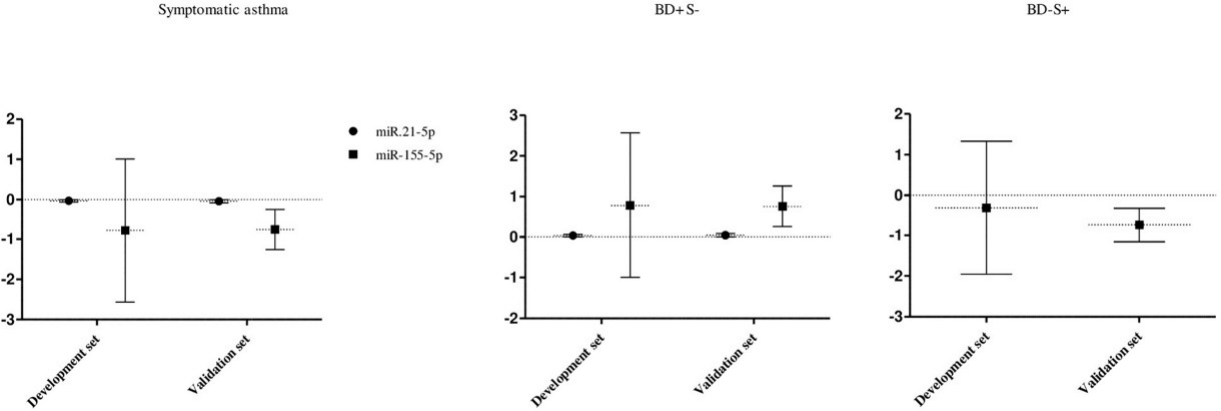
Association between miRNAs and asthma phenotypes. Association between miR-21-5p and miR-155-5p with symptomatic asthma and positive bronchodilation without symptoms in both sets development and validation set. Inverse association between miR-155-5p and negative bronchodilation with asthma symptoms. Asthma: defined based on positive bronchodilation or self-reported medical diagnosis with reported symptoms in the previous year; Symptomatic asthma: defined by “asthma” in a child with current symptoms; BD+S-: Positive bronchodilation without asthma symptoms defined by “asthma” in a child with a current positive bronchodilation test and without symptoms; BD-S+: Negative bronchodilation with asthma symptoms defined by “asthma” in a child with a current negative bronchodilation test and with symptoms.

Regarding asthma phenotypes, miR-21-5p were significantly associated with symptomatic asthma, negatively, and with positive bronchodilation without symptoms, positively, in both sets. Moreover, in validation set miR-155-5p was negatively associated with symptomatic asthma and with negative bronchodilation while it was positively associated with positive bronchodilation without symptoms (Fig 3). A similar trend was detected in development set (Fig 3).

**Fig 4:**
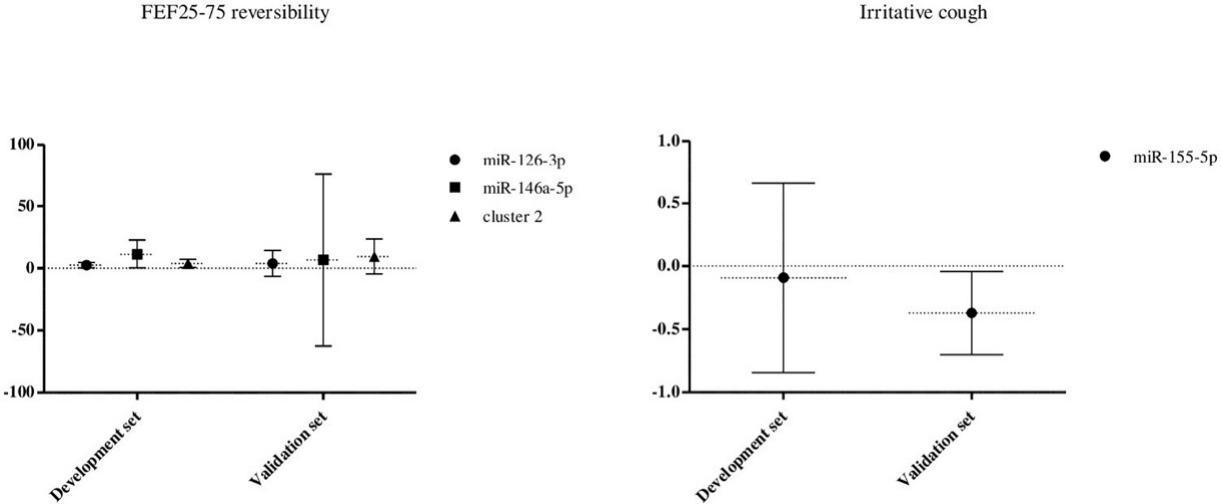
Association between miRNAs and exhaled NO, lung function parameters and respiratory symptoms. Association found between miR-126-3p, miR-146-5p and cluster 2 with higher small airways reversibility and between miR-155-5p with irritative cough. FEF25-75 reversibility: forced expiratory flow middle portion of FVC after bronchodilation.

An association was also found between miR-126-3p, cluster 2 and one of its clustered miRNA, miR-146-5p, with higher small airways reversibility (Fig 4) which was likewise observed in validation set (Fig 4). Lastly, lower levels of miR-155-5p were associated with irritative cough in validation set (Fig 4) whose tendency was similar in development set (Fig 4).

## Discussion

Our study showed that miRNAs can be measured in exhaled breath condensate of school aged children and may be used as potential biomarkers of asthma, assisting asthma endotype establishment. The analysis of miRNAs by principal component analysis revealed the existence of two clusters, one of them associated with children with asthma defined by positive bronchodilation or self-reported medical diagnosis with reported symptoms in the previous year and the other with higher small airways response to salbutamol. Moreover, the analysis of individual miRNAs revealed associations with asthma phenotypes, namely symptomatic and positive bronchodilation without symptoms, lung function parameters and symptoms in the previous three months. Although no significant associations were observed in validation set regarding asthma and small airways response to salbutamol, the results showed a trend that was similar to the development set. Furthermore, the associations between asthma phenotypes and individual miRNAs were confirmed in validation set and it was also observed a similar trend, to the development set, concerning lung function parameters.

To our knowledge, this is the first report addressing exhaled breath condensate miRNAs and asthma in children. However, the present study has some limitations. Firstly it is a cross-sectional study that limits the evaluation through the time, not allowing to establish causal relationships between miRNAs, lung function and asthma diagnosis. Moreover, the use of case-control studies has often been associated with biased estimates of diagnostic accuracy, due to the incorrect sampling of subjects. Accordingly, cohort studies are needed to address additional determinants and variations in exhaled breath condensate miRNAs levels and profiles through the time [34, 35]. However, our study was nested within a well-defined cohort of children where the 'true' disease status was obtained for all with the reference standard. Hence, there was no referral or partial verification bias. Secondly, we did not assess biomarkers, such as blood or sputum eosinophils, which would support on further endotyping children with eosinophilic asthma. In addition, the questionnaire limited the assessment of corticosteroid treatment of children with asthma. Those with well controlled asthma under inhaled steroid treatment may have low exhaled NO, being incorrectly classified as non-eosinophilic asthma. This limitation may be associated with the lack of significance regarding miRNA levels, namely miR-21. Similarly, children with symptoms untreated or treated with inhaled steroids, not considering the exhaled NO levels, were phenotyped as symptomatic. Therefore, children classified as symptomatic may include both mild and severe asthma, which may explain the lack of significant results. Although we fail in evaluating the type and dose of asthma medication that could have driven to differential miRNA expression between asthma phenotypes, we controlled for the currently use of asthma medication. Moreover, in the present study it was not found significant differences (p>0.05) in the miRNAs levels between participants who were under current use of anti-asthma medication and those who were not for each of the analysed phenotypes. Thirdly, there was *a priori* selection of miRNAs based on previous studies, regarding their association with asthma and obesity. Fourthly, our findings may lack external validity as generalization of our observations may not be valid in different age groups. It is also well established that exposure to risk factors at specific time-points may influence the development of asthma, which means that this pathology may change with time [36]. Moreover, given the possibility of remission and relapse it is unclear over how long a period a phenotype should be stable [13]. Lastly, some epigenetic changes can last for many years while others can happen within days and certain associations may differ by age [35]. However, our participants were aged 7 to 12 years, and therefore, it is not expected to find differences regarding phenotype changes. Fifthly, although the present study used classical phenotypes involving traits and features of asthma, requiring subjective choices, while more recent studies have used statistical-data driven clusters, simple phenotypic definitions are preferable to complex ones in both clinical and epidemiological settings [13]. Moreover, despite being in adults, a recent cluster analysis in phenotyping a Portuguese population, identified obesity, lung function and FeNO as important factors for cluster distinction [37] and according to Depner et al., clinical phenotypes were well supported by epidemiologic phenotype definitions identified by latent class analysis [38]. Also, despite numerous validation studies regarding asthma phenotypes in children, there is no phenotype model more valid than others [13]. Sixthly, participants were included in more than one phenotype and had an overlapped association and considering the sample number, phenotyping may reduce the power to detect differences.

On the other hand, our study has also important strengths. Asthma, a heterogeneous condition, was defined based not only on the medical report but also on the presence of symptoms and evidence of airway reversibility. Additionally, in contrast with previous studies, we used different phenotype subgroups allowing the discrimination between children with asthma reducing the heterogeneity within each group. Also, a detailed health data was collected allowing for a relatively unbiased estimate of the prevalence of asthma, allergy sensitization, respiratory symptoms and body mass categories and a high number of EBC samples were analyzed. Furthermore, the collection of EBC is simple, safe, non-invasive and highly repeatable and the technology employed is ideal for miRNA profiling. In addition, we assessed the effects of miRNAs both alone and as a cluster. Lastly, although there was not a complete match between results in both sets, probably due to differences in miRNA levels between development and validation set for asthma phenotypes, the use of an independent validation set confirmed the results obtained from development set, since we observed the same trend in clusters’ characteristics and associations for both populations.

Asthma is an umbrella term to describe a clinical problem, which requires careful multidisciplinary assessment [39]. Furthermore, this disease is heterogeneous and the traditional diagnostic approaches cluster patients according to etiologic diagnosis and treatment. In the present study, we highlighted the asthma heterogeneity, since we have identified children with positive bronchodilation without symptoms and children with asthma defined by positive bronchodilation or medical diagnosis with reported symptoms in the previous year by two different miRNAs. Although it is likely that some mechanisms overlap one or more endotypes, these results reveal that the use of broad definitions can contribute to inconsistent findings between studies.

Several studies on miRNA expression profiling report the importance of miRNAs in the regulation of asthma. However, the cellular phenotype is determined by several epigenetic modifications, like miRNAs, not being one single change in miRNA expression enough to explain the different clinical presentations of asthma. Accordingly, Panganiban *et al*. suggested that expression profiling could be a useful tool to phenotype asthma [10]. While previous studies have focused predominantly on the differences between miRNA levels, individually, among participants with asthma [22, 23, 26]. The present study also assessed the effect of miRNAs clusters, on asthma and its phenotypes, lung function and airway reversibility, exhaled NO and current symptoms.

Expression of some miRNAs has been shown to be different in asthma phenotypes, allowing to distinguish them [5, 40]. However, most studies of miRNAs in asthma are quite heterogeneous, regarding disease definition, biological material assessed, variety of detection technologies used and small sample size for heterogenous phenotypes. Furthermore, miRNAs have different expression profile depending on the cell type and disease status [41]. As in other studies, we performed a prior selection of miRNAs [24, 29]. Six of them were selected because they were deregulated in EBC samples from asthmatics–miR-1248, let7a, miR-155, miR-21, miR-328 and miR-133a [22]. Additionally, five others claimed to be related to inflammatory responses–miR-126, miR-145, miR-146 and miR-328 [6]—or obesity–miR-423 [42]–and were therefore included. Since asthma and obesity are characterized by chronic inflammation, it is likely that miRNAs might be deregulated in these diseases, modulating immune cells that may be found in EBC.

Exhaled breath condensate has been developed as an alternative to induce sputum [1]. However, detection and quantification of miRNAs in this body fluid has been poorly explored. Pinkerton *et al*. reported that miR-21, miR-133, miR-155 and miR-328 in EBC were significantly lower in asthmatic adults [22]. Although, we did not observe significant differences in the levels of miR-21-5p, miR-133a-3p and miR-155-5p between children with and without asthma or within asthma phenotypes, miR-328-3p was downregulated in both development and validation set, being significant in the latter. However, one of the clusters encompassing miR-133a-3p and miR328-3p, as well as, miR-133a-3p alone, were positively associated with asthma in children. In addition, miR-21-5p was identified as biomarkers of airway reversibility being significantly associated with positive bronchodilation without symptoms, despite being negatively associated with symptomatic asthma. Recently, Ong *et al*., found that some miRNAs were lower expressed in the airways of healthy individuals with increasing age [43]. Although we are studying children with asthma, these differences in findings may be due to studies heterogeneity related with age group as well as sample size, and, importantly, dissimilar asthma definitions.

Many miRNAs have anti-inflammatory functions and some of them have been shown to be deregulated in asthmatic children [22]. Our study provides new evidence and further supports the value of using miRNAs for the identification of asthma traits, allowing for a more precise approach on targeted therapy. Notably, our findings showed that miR-126-3p, miR-133a-3p and miR-145-5p identified airway reversibility or self-reported medical diagnosis with reported symptoms in the previous year. The results obtained by Lacedonia *et al*. showed that miRNA-145 was upregulated in the supernatant of adults patients with asthma which corroborates our findings [7]. Furthermore we found that higher levels of miR-146a-3p were associated with higher small airway reversibility, making this miRNA a plausible biomarker for the airway trait when endotyping asthma. Moreover, in validation set, miR-155-5p additionally emerge as a potential biomarker of airways being significantly decreased in symptomatic asthma and in negative bronchodilation with asthma symptoms. Accordingly, it was found that miR-155 was significantly increased in the plasma of children with asthma [44]. Altogether, our results indicate that miRNAs, individually or clustered, may identify asthma and uncover features and traits of asthma in school aged children.

The lungs have been demonstrated to have a very distinctive miRNA profile [45] but these molecules have the potential to target hundreds of messenger RNAs, creating a challenge in identifying their functional roles. Furthermore, abnormal expression of miRNAs may contribute to the development and progression of asthma. Several mechanisms may be associated with this deregulation. It was proposed that miRNAs were predicted to regulate Th2 cytokines receptors, suggesting that deregulation of this pathway could have implications on the expression and signaling of Th2 cytokines [22].For instance, miR-21 appears to promote Th2 and attenuates Th1 pathway while the upregulation of miR-155 in mild-asthmatics seems to suppress cytokine expression induced by IL-13 [46]. The association found between miR-21 and positive bronchodilation without symptoms could be explained by the role of this miR-21 in negatively regulate IL-12, important for the regulation of Th1/Th2 balance since a decreased expression of this IL induces an excessive Th2 response [47]. In fact, miR-21 has been linked to eosinophilic inflammation but we were not able to find a significant association with eosinophilic asthma phenotype. This may be explained through the use of different markers (exhaled NO levels versus eosinophil percentage). On the other hand, miR-155 was negatively associated with symptomatic asthma phenotype as well as with negative bronchodilation with symptoms that may be explained by the fact that this miRNA is able to regulate the Th2 inflammation through a downregulation of the secretion of IL-4, IL-5 and IL-13 by Th2 cells [48].

Therefore, the expression of particular miRNAs, namely miR-133a-3p and miR-126-3p, may be induced in immune cells that were stimulated by pro-inflammatory cytokines, such as TNF-α. Previous studies showed that miR-126 increases GATA-3 expression in T cells, in an indirect way that could promote a Th2 response and are thought to increase the levels of the IL-13, suggesting to be associated with an excessive activation of Th2 cells in asthmatic children [49]. Additionally, miR-133 seems to regulate the IL-13, which suggests that dysregulation of this pathway could have major implications on the expression and signalling of Th2 cytokines [22]. Moreover, miR-145 was demonstrated that it may target RUNX3, which can attenuate GATA-3, regulator of the Th2 differentiation and necessary for immune responses mediated by Th2. Therefore, higher levels of this miRNA led to an inhibition of RUNX3 expression, which indicates that miR-145 is involved in the pathogenesis of asthma through the regulation of Th1/Th2 balance by targeting RUNX3 [50]. These mechanisms are consistent with the association found between miR-126 and mIR-133 with asthma, defined by a positive bronchodilation or medical diagnosis with asthma symptoms in the previous 12 months. Additionally, others miRNAs may be associated with negative feedback mechanism that limit the inflammatory responses by inhibiting NF-kβ [51], such as miR-146a-5p, thereby resulting in suppression of expression of specific miRNAs and reducing the inflammatory processes in the airways.

Several biomarkers have been studied in exhaled breath condensate rather than miRNAs. Rufo *et al*. developed a hierarchical model through the use of an electronic nose that analyzed exhaled breath condensate VOCs from children, being able to discriminate children with asthma, defined by medical diagnosis, and those with persistent asthma, based on corticosteroid therapy [52]. Another study conducted in children, revealed that 8 volatile organic compounds were increased in EBC of children with asthma [53]. Components associated with retinoic acid, adenosine and vitamin D present in EBC were also indicated as relevant for the discrimination both between children with and without severe asthma [54]. Moreover, components involved in methane, pyruvate, and glyoxylate and dicarboxylate metabolic pathways were specifically found to distinguish patients with both asthma and obesity from the other participants [55]. These studies showed that breathomics are able to discriminate different asthma phenotypes, being in accordance with our results.

In conclusion, we showed that the measurement of miRNAs in EBC is feasible, simple, safe and non-invasive. This work demonstrates that specific miRNAs, which were previously described as being deregulated in children with asthma, as well as EBC miRNA profiles, show potential to identify asthma. Furthermore, our analyses reveal significant associations between miRNAs and specific traits of asthma such as lung function and airway reversibility. In all, these results indicate that using EBC microRNAs as biomarkers of asthma and associated lung function impairment may assist asthma endotype establishment on the way to a more personalized treatment.

## Supporting information

S1 Fig Principal component analysis. (DOCX)

S1 Table Associations between miRNAs and asthma and asthma phenotypes in development set. (DOCX)

S2 Table Associations between miRNAs and asthma and asthma phenotypes in validation set. (DOCX)

S3 Table Associations between miRNAs, exhaled NO, lung function and current symptoms in development set. (DOCX)

S4 Table Associations between miRNAs, exhaled NO, lung function and current symptoms in validation set. (DOCX)
