# Supplementary material for: Development and validation of exhaled breath condensate microRNAs to identify and endotype asthma in children
Source: PLoS One. 2019 Nov 8;14(11):e0224983. doi: 10.1371/journal.pone.0224983 (PMC6839869; doi:10.1371/journal.pone.0224983)
Supplement: S1 Fig — (DOCX) [file pone.0224983.s001.docx]

S1 Fig. Variables Principal component analysis


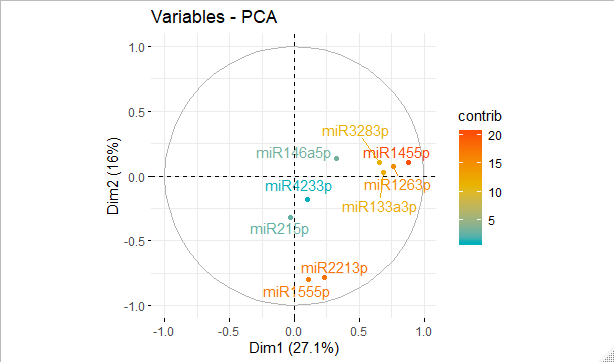


Positive correlated variables point to the same side of the plot. Negative correlated variables point to opposite sides of the graph.
