# Supplementary material for: Development and validation of exhaled breath condensate microRNAs to identify and endotype asthma in children
Source: PLoS One. 2019 Nov 8;14(11):e0224983. doi: 10.1371/journal.pone.0224983 (PMC6839869; doi:10.1371/journal.pone.0224983)
Supplement: S1 Table — (DOCX) [file pone.0224983.s002.docx]

S1 Table. Associations between miRNAs and asthma and asthma phenotypes in development set

|  | *Asthma* | | *Allergic asthma* | | *Eosinophilic asthma* | |
| --- | --- | --- | --- | --- | --- | --- |
|  | Crude model | Adjusted model^a^ | Model 0 | Model 1^b^ | Model 0 | Model 1^c^ |
|  | β (95% CI) | β (95% CI) | β (95% CI) | β (95% CI) | β (95% CI) | β (95% CI) |
| *miR-21-5p* | -0.01 (-0.02; 0.01) | -4.00x10^-3^ (-0.01; 0.01) | -5.00x10^-3^ (-0.04; 0.03) | 4.00x10^-3^ (-0.04; 0.04) | -0.01 (-0.05; 0.02) | -0.02 (-0.05; 0.02) |
| *miR-126-3p* | **0.08 (0.01; 0.14)** | **0.08 (0.03; 0.13)** | -0.02 (-0.09; 0.06) | -0.02 (-0.10; 0.06) | -0.04 (-0.10; 0.03) | -0.03 (-0.10; 0.05) |
| *miR-133a-3p* | **0.18 (0.1; 0.34)** | **0.16 (0.03; 0.30)** | -0.13 (-0.32; 0.06) | -0.10 (-0.30; 0.11) | -0.11 (-0.29; 0.07) | -0.09 (-0.27; 0.10) |
| *miR-145-5p* | 0.04 (-6x10-3; 0.08) | **0.04 (0.01; 0.07)** | -0.03 (-0.07; 0.02) | -0.02 (-0.07; 0.02) | -0.02 (-0.07; 0.02) | -0.01 (-0.06; 0.03) |
| *miR-146a-5p* | 0.27 (-0.08; 0.62) | 0.25 (-0.03; 0.52) | 0.06 (-0.32; 0.44) | 0.04 (-0.35; 0.43) | -0.07 (-0.42; 0.28) | -0.05 (-0.40; 0.31) |
| *miR-155-5p* | -0.43 (-1.15; 0.30) | -0.09 (-0.67; 0.50) | -1.06 (-3.16; 1.03) | -1.29 (-3.49; 0.91) | -0.69 (-2.64; 1.25) | -0.26 (-2.35; 1.83) |
| *miR-221-3p* | 0.05 (-0.03; 0.13) | 0.06 (-6x10^-3^; 0.12) | -0-06 (-0.14; 0.03) | -0.04 (-0.13; 0.04) | -0.03 (-0.11; 0.05) | -0.02 (-0.10; 0.06) |
| *miR-328-3p* | 0.02 (-0.03; 0.07) | 0.02 (-0.02; 0.06) | -0.04 (-0.10; 0.02) | -0.03 (-0.09; 0.04) | -0.04 (-0.10; 0.01) | -0.03 (-0.09; 0.03) |
| *miR-423-3p* | 0.05 (-0.14; 0.23) | 0.11 (-0.04; 0.26) | 0.12 (-0.09; 0.32) | 0.10 (-0.12; 0.32) | -0.05 (-0.24; 0.14) | -0.06 (-0.26; 0.14) |
| Cluster 1 | **0.11 (3.00x10^-3^; 0.21)** | **0.12 (0.04; 0.20)** | -0.06 (-0.18; 0.05) | -0.05 (-0.18; 0.07) | -0.07 (-0.18; 0.03) | -0.05 (-0.16; 0.06) |
| Cluster 2 | 0.03 (-0.08; 0.14) | 0.04 (-0.05; 0.12) | 0.07 (-0.04; 0.18) | 0.05 (-0.07; 0.17) | 2.00x10^-3^ (-0.10; 0.11) | -5.00x10^-3^ (-0.11; 0.10) |
|  | *Obese asthma* | | *Persistent asthma* | | *Symptomatic asthma* | |
|  | Model 0 | Model 1^d^ | Model 0 | Model 1^e^ | Model 0 | Model 1^a^ |
| *miR-21-5p* | -0.02 (-0.06; 0.02) | 0.26 (-0.04; 0.01) | -5.00x10^-3^ (-0.04; 0.03) | -2.00x10^-3^ (-0.04; 0.04) | -0.02 (-0.06; 0.02) | **-0.03 (-0.06; -2.00x10^-3^)** |
| *miR-126-3p* | 0.02 (-0.05; 0.09) | 1.00x10^-3^ (-0.05; 0.05) | -0.03 (-0.10; 0.04) | -0.05 (-0.12; 0.03) | -0.04 (-0.11; 0.03) | -0.1 (0.08; 0.05) |
| *miR-133a-3p* | -0.02 (-0.21; 0.17) | -0.04 (-0.17; 0.09) | -0.05 (-0.24; 0.14) | -0.04 (-0.25; 0.16) | -0.10 (-0.29; 0.08) | -0.11 (-0.27; 0.05) |
| *miR-145-5p* | -0.02 (-0.06; 0.03) | -6.00x10^-3^ (-0.04; 0.02) | -0.03 (-0.07; 0.02) | -0.02 (-0.07; 0.02) | -0.04 (-0.08; 7.00x10^-3^) | -0.02 (-0.06; 0.02) |
| *miR-146a-5p* | 0.02 (-0.35; 0.39) | 0.07 (-0.16; 0.31) | -0.09 (-0.46; 0.28) | -0.07 (-0.45; 0.31) | -0.13 (-0.49; 0.24) | -0.01 (-0.32; 0.30) |
| *miR-155-5p* | 1.14 (-0.89; 3.17) | -0.16 (-1.56; 1.24) | -1.24 (-3.17; 0.89) | -1.46 (-3.57; 0.64) | -1.55 (-3.53; 0.42) | -0.78 (-2.57; 1.01) |
| *miR-221-3p* | -3.00x10^-3^ (-0.09; 0.08) | -0.01 (-0.07; 0.04) | -0.02 (-0.11; 0.06) | -0.02 (-0.11; 0.06) | -0.05 (-0.13; 0.03) | -0.05 (-0.12; 0.02) |
| *miR-328-3p* | 8.00x10^-3^ (-0.05; 0.07) | -9.00x10^-3^ (-0.05; 0.03) | -4.00x10^-3^ (-0.06; 0.06) | -3.92x10^-4^ (-0.07; 0.07) | -0.01 (-0.07; 0.05) | -0.02 (-0.07; 0.04) |
| *miR-423-3p* | -0.08 (-0.28; 0.12) | 0.02 (-0.12; 0.16) | -0.12 (-0.32; 0.08) | -0.12 (-0.33; 0.09) | -0.13 (-0.32; 0.07) | -0.10 (-0.19; 0.17) |
| Cluster 1 | -8.00x10^-3^ (-0.12; 0.11) | -0.01 (-0.09; 0.06) | -0.05 (-0.17; 0.06) | -0.05 (-0.17; 0.07) | -0.08 (-0.19; 0.03) | -0.06 (-0.15; 0.04) |
| Cluster 2 | -1.00x10^-3^ (-0.11; 0.11) | 0.02 (-0.05; 0.10) | -0.03 (-0.14; 0.08) | -0.03 (-0.14; 0.09) | -0.02 (-0.13; 0.09) | 0.03 (-0.07; 0.12) |
|  | *BD+S+* | | *BD+S-* | | *BD-S+* | |
|  | Model 0 | Model 1^a^ | Model 0 | Model 1^a^ | Model 0 | Model 1^a^ |
| *miR-21-5p* | -0.01 (-0.04; 0.02) | -0.03 (-0.05; 2.00x10^-3^) | 0.02 (-0.02; 0.06) | **0.03 (2.00x10^-3^; 0.06)** | -6.00x10^-3^ (-0.05; 0.03) | -1.00x10^-3^ (-0.03; 0.03) |
| *miR-126-3p* | -0.03 (-0.08; 0.03) | -0.03 (-0.09; 0.03) | 0.04 (-0.03; 0.11) | 0.01 (-0.05; 0.08) | -0.01 (-0.08; 0.06) | 0.02 (-0.04; 0.08) |
| *miR-133a-3p* | -0.09 (-0.23; 0.06) | -0.14 (-0.29; 8.00x10^-3^) | 0.10 (-0.08; 0.29) | 0.11 (-0.05; 0-27) | -0.02 (-0.21; 0.18) | 0.03 (-0.12; 0.18) |
| *miR-145-5p* | -0.02 (-0.05; 0.02) | -0.02 (-0.06; 0.01) | 0.04 (-7.00x10^-3^; 0.08) | 0.02 (-0.02; 0.06) | -0.02 (-0.06; 0.03) | 1.00x10^-3^ (-0.03; 0.04) |
| *miR-146a-5p* | -0.09 (-0.38; 0.20) | -0.06 (-0.35; 0.24) | 0.13 (-0.24; 0.49) | 0.01 (-0.30; 0.32) | -0.04 (-0.42; 0.34) | 0.05 (-0.23; 0.33) |
| *miR-155-5p* | -0.43 (-2.05; 1.19) | 0.47 (-2.19; 1.24) | 1.55 (-0,42; 3.53) | 0.78 (-1.00; 2.57) | -1.12 (-3.22; 0.98) | -0.31 (-1.95; 1.33) |
| *miR-221-3p* | -0..02 (-0.09; 0.04) | -0.04 (-0.10; 0.03) | 0.05 (-0.03; 0.13) | 0.05 (-0.02; 0.12) | -0.03 (-0.11; 0.06) | -0.01 (-0.07; 0.05) |
| *miR-328-3p* | -0.03 (-0.07; 0.02) | -0.04 (-0.09; 0.01) | 0.01 (-0.05; 0.07) | 0.02 (-0.04; 0.07) | 0.01 (-0.05; 0.08) | 0.02 (-0.03; 0.07) |
| *miR-423-3p* | -0.04 (-0.19; 0.12) | -0.05 (-0.21; 0.12) | 0.13 (-0.07; 0.32) | 0.01 (-0.17; 0.19) | -0.09 (-0.30; 0.11) | 0.04 (-0.12; 0.20) |
| Cluster 1 | -0.05 (-0.14; 0.03) | -0.08 (-0.17; 0.02) | 0.08 (-0.03; 0.19) | 0.06 (-0.04; 0.15) | -0.03 (-0.15; 0.09) | 0.02 (-0.07; 0.11) |
| Cluster 2 | -4.00x10^-3^ (-0.09; 0.08) | 0.01 (-0.08; 0.10) | 0.02 (-0.09; 0.13) | -0.03 (-0.12; 0.07) | -0.01 (-0.13; 0.10) | 0.02 (-0.07; 0.10) |

Cluster 1: typified by miR-126-3p, miR-133a-3p, miR-145-5p, miR-221-3p and miR-328-3p

Cluster 2: typified by miR-146a-5p and miR-423-3p

Asthma: defined based on positive bronchodilation or self-reported medical diagnosis with reported symptoms in the previous year

Allergic asthma: defined by “asthma” in a child with positive skin prick test

Eosinophilic asthma: defined by “asthma” in a child with exhaled nitric oxide above 35 ppb

Obese asthma: defined by “asthma” in an overweight or obese child

Persistent asthma: defined by “asthma” in a child currently using anti-asthma medication

Symptomatic asthma: defined by “asthma” in a child with current symptoms

BD+S+: Positive bronchodilation with asthma symptoms defined by “asthma” in a child with a current positive bronchodilation test and symptoms

BD+S-: Positive bronchodilation without asthma symptoms defined by “asthma” in a child with a current positive bronchodilation test and without symptoms

BD-S+: Negative bronchodilation with asthma symptoms defined by “asthma” in a child with a current negative bronchodilation test and with symptoms

^a^: adjusted for: age, sex, exhaled NO, atopy, body mass categories according to CDC and currently using anti-asthma medication

^b^: adjusted for: age, sex, exhaled NO, body mass categories according to CDC and currently using anti-asthma medication

^c^: adjusted for: age, sex, atopy, body mass categories according to CDC and currently using anti-asthma medication

^d^: adjusted for: age, sex, exhaled NO, atopy and currently using anti-asthma medication

^e^: adjusted for: age, sex, exhaled NO, atopy and body mass categories according to CDC

Significant differences **in bold**
