# Supplementary material for: Development and validation of exhaled breath condensate microRNAs to identify and endotype asthma in children
Source: PLoS One. 2019 Nov 8;14(11):e0224983. doi: 10.1371/journal.pone.0224983 (PMC6839869; doi:10.1371/journal.pone.0224983)
Supplement: S2 Table — (DOCX) [file pone.0224983.s003.docx]

S2 Table. Associations between miRNAs and asthma and asthma phenotypes in validation set

|  | *Asthma* | | *Allergic asthma* | | *Eosinophilic asthma* | |
| --- | --- | --- | --- | --- | --- | --- |
|  | Model 0 | Model 1^a^ | Model 0 | Model 1^b^ | Model 0 | Model 1^c^ |
|  | β (95% CI) | β (95% CI) | β (95% CI) | β (95% CI) | β (95% CI) | β (95% CI) |
| *miR-21-5p* | -7.00x10^-3^ (-0.03; 0.01) | -6.00x10^-3^ (-0.02; 0.01) | -0.32 (-0.08; 0.02) | -0.04 (-0.09; 6.00x10^-3^) | -9.00x10-3 (-0.05; 0.03) | -4.00x10^-3^ (-0.05; 0.04) |
| *miR-126-3p* | 0.03 (-0.07; 0.13) | 0.05 (-0.04; 0.14) | -0.10 (-0.25; 0.05) | -0.08 (-0.24; 0.09) | -0.06 (-019; 0.07) | -0.06 (-0.18; 0.06) |
| *miR-133a-3p* | 6.00x10-3 (-0.13; 0.14) | 0.01 (-0.14; 0.12) | 0.05 (-0.23; 0.34) | -0.03 (-0.33; 0.26) | -0.07 (-0.31; 0.17) | -0.06 (-0.28; 0.16) |
| *miR-145-5p* | -3.00x10-3 (-0.05; 0.04) | 3.00x10^-3^ (-0.04; 0.05) | -5.00x10-3 (-0.09; 0.08) | -0.03 (-0.11; 0.06) | -0.03 (-0.10; 0.04) | 2.00x10^-3^ (-0.07; 0.07) |
| *miR-146a-5p* | -0.23 (-1.51; 1.05) | 0.41 (-0.75; 1.58) | -2.23 (-4.68; 0.22) | -1.16 (-4.29; 1.97) | -0.90 (-3.05; 1.25) | -1.24 (-3.46; 0.99) |
| *miR-155-5p* | -0.10 (-0.45; 0.25) | -0.06 (-0.38; 0.27) | 0.38 (-0.42; 1.17) | 0.16 (-0.66; 0.98) | -0.09 (-0.76; 0.59) | -0.10 (-0.72; 0.52) |
| *miR-221-3p* | 0.15 (-0.04; 0.34) | 0.11 (-0.07; 0.28) | 0.08 (-0.24; 0.40) | -7.00x10^-3^ (-0.34; 0.33) | -0.09 (-0.36; 0.18) | -0.04 (-0.29; 0.20) |
| *miR-328-3p* | 4.00x10-3 (-0.07; 0.08) | -0.03 (-0.10; 0.04) | **0.16 (0.04; 0.27)** | 0.10 (-0.05; 0.25) | -0.02 (-0.13; 0.09) | -0.02 (-0.14; 0.10) |
| *miR-423-3p* | 0.20 (-0.32; 0.72) | 0.19 (-0.28; 0.65) | -0.10 (-0.97; 0.77) | 0.06 (-0.83; 0.94) | 0.12 (-0.61; 0.85) | 0.04 (-0.63; 0.71) |
| Cluster 1 | 0.02 (-0.07; 0.12) | 0.02 (-0.07; 0.11) | 0.02 (-0.14; 0.18) | -0.04 (-0.21; 0.13) | -0.08 (-0.21; 0.06) | -0.05 (-0.18; 0.08) |
| Cluster 2 | 0.01 (-0.08; 0.11) | 0.04 (-0.05; 0.13) | -0.18 (-0.37; 0.01) | -0.11 (-0.33; 0.11) | -0.04 (-0.21; 0.13) | -0.05 (-0.22; 0.13) |
|  | *Obese asthma* | | *Persistent asthma* | | *Symptomatic asthma* | |
|  | Model 0 | Model 1^d^ | Model 0 | Model 1^e^ | Model 0 | Model 1^a^ |
| *miR-21-5p* | 7.00x10^-3^ (-0.04; 0.05) | 1.70x10^-4^ (-0.06; 0.06) | -6.00x10^-3^ (-0.06; 0.04) | 5.00x10^-3^ (-0.05; 0.06) | -0.02 (-0.07; 0.02) | **-0.04 (-0.08; -4.00x10^-3^)** |
| *miR-126-3p* | 0.05 (-0.10; 0.20) | 0.02 (-0.16; 0.20) | -0.10 (-0.25; 0.05) | 0.02 (-0.14; 0.17) | -0.11 (-0.26; 0.04) | -0.04 (-0.16; 0.09) |
| *miR-133a-3p* | 0.03 (-0.25; 0.31) | 0.04 (-0.26; 0.34) | 0.11 (-0.17; 0.40) | 0.08 (-0.18; 0.35) | -0.10 (-0.38; 0.19) | -0.19 (-0.39; 3.00x10^-3^) |
| *miR-145-5p* | 0.03 (-0.05; 0.11) | 0.03 (-0.06; 0.13) | -7.00x10^-3^ (-0.09; 0.08) | -0.02 (-0.10; 0.07) | -0.03 (-0.12; 0.05) | -0.05 (-0.12; 8x10^-3^) |
| *miR-146a-5p* | 0.77 (-1.70; 3.25) | 0.36 (-2.86; 3.59) | -2.25 (-4.71; 0.21) | -0.13 (-3.08; 2.81) | -0.63 (-3.21; 1.95) | 1.52 (-0.74; 3.78) |
| *miR-155-5p* | -0.28 (-1.04; 0.49) | -0.26 (-1.10; 0.59) | 0.30 (-0.51; 1.10) | 0.21 (-0.53; 0.96) | -0.54 (-1.32; 0.24) | **-0.76 (-1.26; -0.25)** |
| *miR-221-3p* | 0.07 (-0.24; 0.38) | 0.10 (-0.25; 0.44) | 0.13 (-0.19; 0.45) | 0.02 (-0.28; 0.33) | 0.07 (-0.25; 0.39) | -0.03 (-0.27; 0.22) |
| *miR-328-3p* | -0.06 (-0.18; 0.06) | -0.05 (-0.22; 0.11) | **0.13 (7.00x10^-3^; 0.25)** | 0.09 (-0.05; 0.23) | 0.09 (-0.04; 0.22) | 0.01 (-0.11; 0.13) |
| *miR-423-3p* | 0.58 (-0.23; 1.38) | 0.63 (-0.23; 1.49) | -8.00x10^-3^ (-0.89; 0.87) | 0.02 (-0.80; 0.83) | -0.02 (-0.89; 0.85) | -0.06 (-0.71; 0.59) |
| Cluster 1 | 0.03 (-0.12; 0.19) | 0.03 (-0.15; 0.20) | 0.03 (-0.13; 0.20) | 0.04 (-0.12; 0.19) | -0.07 (-0.23; 0.09) | -0.11 (-0.23; 1.00x10^-3^) |
| Cluster 2 | 0.14 (-0.04; 0.33) | 0.14 (-0.08; 0.37) | -0.13 (-0.33; 0.07) | -0.02 (-0.23; 0.19) | -0.07 (-0.27; 0.14) | 6.00x10^-3^ (-0.16; 0-18) |
|  | *BD+S+* | | *BD+S-* | | *BD-S+* | |
|  | Model 0 | Model 1^a^ | Model 0 | Model 1^a^ | Model 0 | Model 1^a^ |
| *miR-21-5p* | 4.00x10^-3^ (-0.03; 0.03) | -0.01 (-0.05; 0.02) | 0.02 (-0.02; 0.07) | **0.04 (4x10^-3^; 0.08)** | -0.03 (-0.08; 0.02) | -0.03 (-0.06; 5.00x10^-3^) |
| *miR-126-3p* | -0.03 (-0.12; 0.07) | -0.07 (-0.17; 0.04) | 0.11 (-0.04; 0.26) | 0.04 (-0.09; 0.16) | -0.09 (-0.24; 0.06) | 0.03 (-0.08; 0.14) |
| *miR-133a-3p* | -0.07 (-0.24; 0.10) | -0.07 (-0.26; 0.11) | 0.10 (-0.19; 0.38) | 0.19 (-3.00x10^-3^; 0.39) | -0.03 (-0.31; 0.26) | -0.12 (-0.30; 0.06) |
| *miR-145-5p* | -0.01 (-0.06; 0.04) | -0.03 (-0.09; 0.03) | 0.03 (-0.05; 0.12) | 0.05 (-8.00x10^-3^; 0.12) | -0.02 (-0.10; 0.06) | -0.02 (-0.08; 0.03) |
| *miR-146a-5p* | **1.60 (0.19; 3.02)** | 1.54 (-0.40; 3.48) | 0.63 (-1.95; 3.21) | -1.52 (-3.78; 0.74) | -2.23 (-4.68; 0.22) | -0.02 (-2.10; 2.06) |
| *miR-155-5p* | -0.08 (-0.56; 0.40) | -0.02 (-0.54; 0.51) | 0.54 (-0.24; 1.32) | **0.76 (0.25; 1.26)** | -0.46 (-1.25; 0.33) | **-0.74 (-1.16; -0.32)** |
| *miR-221-3p* | -0.05 (-0.24; 0.14) | -0.03 (-0.24; 0.19) | -0.07 (-0.40; 0.25) | 0.03 (-0.22; 0.27) | 0.12 (-0.20; 0.44) | -3-37x10^-4^ (-0.22; 0.22) |
| *miR-328-3p* | -0.03 (-0.11; 0.04) | -5.00x10^-3^ (-0.11; 0.10) | -0.09 (-0.22; 0.04) | -0.01 (-0.13; 0.11) | **0.12 (1.00x10^-3^; 0.24)** | 0.02 (-0.09; 0.12) |
| *miR-423-3p* | 0.07 (-0.45; 0.58) | 0.04 (-0.53; 0.61) | 0.02 (-0.85; 0.89) | 0.06 (-0.59; 0.71) | -0.08 (-0.96; 0.78) | -0.11 (-0.68; 0.47) |
| Cluster 1 | -0.05 (-0.15; 0.04) | -0.07 (-0.18; 0.03) | 0.07 (-0.09; 0.23) | 0.11 (-1.00x10^-3^; 0.23) | -0.02 (-0.18; 0.14) | -0.04 (-0.15; 0.07) |
| Cluster 2 | 0.07 (-0.04; 0.19) | 0.03 (-0.12; 0.18) | 0.07 (-0.14; 0.27) | 6.00x10^-3^ (-0.18; 0.16) | -0.14 (-0.34; 0.06) | -0.03 (-0.17; 0.12) |

Cluster 1: typified by miR-126-3p, miR-133a-3p, miR-145-5p, miR-221-3p and miR-328-3p

Cluster 2: typified by miR-21-5p, miR-146a-5p and miR-423-3p

Asthma: defined based on positive bronchodilation or self-reported medical diagnosis with reported symptoms in the previous year

Allergic asthma: defined by “asthma” in a child with positive skin prick test

Eosinophilic asthma: defined by “asthma” in a child with exhaled nitric oxide above 35 ppb

Obese asthma: defined by “asthma” in an overweight or obese child

Persistent asthma: defined by “asthma” in a child currently using anti-asthma medication

Symptomatic asthma: defined by “asthma” in a child with current symptoms

BD+S+: Positive bronchodilation with asthma symptoms defined by “asthma” in a child with a current positive bronchodilation test and symptoms

BD+S-: Positive bronchodilation without asthma symptoms defined by “asthma” in a child with a current positive bronchodilation test and without symptoms

BD-S+: Negative bronchodilation with asthma symptoms defined by “asthma” in a child with a current negative bronchodilation test and with symptoms

Model 0: crude model.

^a^: adjusted for: age, sex, exhaled NO, atopy, body mass categories according to CDC and currently using anti-asthma medication

^b^: adjusted for: age, sex, exhaled NO, body mass categories according to CDC and currently using anti-asthma medication

^c^: adjusted for: age, sex, atopy, body mass categories according to CDC and currently using anti-asthma medication

^d^: adjusted for: age, sex, exhaled NO, atopy and currently using anti-asthma medication

^e^: adjusted for: age, sex, exhaled NO, atopy and body mass categories according to CDC

Significant differences **in bold**
