# Supplementary material for: Development and validation of exhaled breath condensate microRNAs to identify and endotype asthma in children
Source: PLoS One. 2019 Nov 8;14(11):e0224983. doi: 10.1371/journal.pone.0224983 (PMC6839869; doi:10.1371/journal.pone.0224983)
Supplement: S3 Table — (DOCX) [file pone.0224983.s004.docx]

S3 Table. Associations between miRNAs, exhaled NO, lung function and current symptoms in development set

|  | *exhaled NO* | | *FVC (% predicted)* | |
| --- | --- | --- | --- | --- |
|  | Model 0 | Model 1^a^ | Model 0 | Model 1^b^ |
|  | β (95% CI) | β (95% CI) | β (95% CI) | β (95% CI) |
| *miR-21-5p* | -5.00x10^-3^ (-0.01; 5.00x10^-3^) | -4.00x10^-3^ (-0.01; 5.00x10^-3^) | 0.10 (-0.35; 0.55) | 0.06 (-0,34; 0.46) |
| *miR-126-3p* | -0.01 (-0.6; 0.04) | -0.03 (-0.08; 0.02) | -0.56 (-2.91; 1.79) | -0.13 (-2.28; 2.02) |
| *miR-133a-3p* | -0.05 (-0.17; 0.08) | -0.08 (-0.21; 0.04) | 5.51 (-0.40; 11.4) | 3.75 (-1.63; 9.13) |
| *miR-145-5p* | -0.01 (-0.05; 0.02) | -0.02 (-0.05; 9.00x10^-3^) | **1.48 (2.00x10^-3^; 2.96)** | 1.22 (-0.07; 2.51) |
| *miR-146a-5p* | -3.00x10^-3^ (-0.26; 0.26) | -0.08 (-0.34; 0.17) | -1.40 (-14.1; 11.3) | -1.29 (-12.3; 9.76) |
| *miR-155-5p* | -0.25 (-0.79; 0.28) | -0.11 (-0.64; 0.41) | -18.9 (-46.0; 8.18) | -9.61 (-32.8; 13.6) |
| *miR-221-3p* | -0.02 (-0.07; 0.04) | -0.02 (-0.08; 0.04) | 2.27 (-0.44; 4.98) | 2.27 (-0.08;4.61) |
| *miR-328-3p* | -0.02 (-0.06; 0.01) | -0.03 (-0.06; 7.00x10^-3^) | 0.09 (-1.57; 1.76) | 0.32 (-1.12; 1.77) |
| *miR-423-3p* | -0.04 (-0.17; 0.10) | -0.77 (-0.21; 0.06) | -2.91 (-9.43; 3.61) | -0.67 (-6.51; 5.17) |
| Cluster 1 | -0.04 (-0.12; 0.04) | -0.07 (-0.15; 0.01) | 1.99 (-1.77; 5.75) | 2.08 (-1.27; 5.43) |
| Cluster 2 | 9.00x10^-3^ (-0.07; 0.09) | -0.01 (-0.09; 0.07) | -2.76 (-6.46; 0.95) | -1.83 (-5.08; 1.41) |
|  | *FEV1 (% predicted)* | | *FEF25-75 (% predicted)* | |
|  | Model 0 | Model 1^b^ | Model 0 | Model 1^b^ |
| *miR-21-5p* | 0.09 (-0.36; 0.53) | 0.10 (-0.29; 0.49) | -0.11 (-0.87; 0.66) | 0.08 (-0.71; 0.87) |
| *miR-126-3p* | 0.31 (-2.01; 2.61) | 0.57 (-1.52; 2.67) | 3.55 (-0.38; 7.48) | 3.02 (-1.16; 7.20) |
| *miR-133a-3p* | 4.41 (-1.43; 10.3) | 2.07 (-3.23; 7.38) | 1.49 (-8.81; 11.8) | -0.86 (-11.6; 9.92) |
| *miR-145-5p* | 0.92 (-0.55; 2.40) | 0.61 (-0.67; 1.90) | -0.29 (-2.88; 2.30) | -0.54 (-3.15; 2.07) |
| *miR-146a-5p* | 1.46 (-11.0; 13.9) | 0.73 (-10.1; 11.5) | 10.2 (11.3; 31.7) | 6.01 (-15.8; 27.8) |
| *miR-155-5p* | -15.4 (-42.1; 11.2) | -4.48 (-27.3; 18.3) | -1.71 (-48.5; 45.0) | 7.57 (-38.5; 53.6) |
| *miR-221-3p* | 1.16 (-1.53; 3.85) | 0.97 (-1.36; 3.31) | -1.45 (-6.15; 3.24) | -1.95 (-6.68; 2.77) |
| *miR-328-3p* | -0.08 (-1.71; 1.56) | 0.13 (-1.29; 1.54) | -0.13 (-2.97; 2.71) | -0.18 (-3.04; 2.68) |
| *miR-423-3p* | -1.39 (-7.81; 5.03) | 0.99 (-4.71; 6.68) | 5.98 (-5.10; 17.1) | 6.86 (-4.55; 18.3) |
| Cluster 1 | 1.57 (-2.13; 5.27) | 1.38 (-1.91; 4.67) |  | 0.54 (-6.16; 7.23) |
| Cluster 2 | -1.11 (-4.79; 2.57) | -0.26 (-3.45; 2.94) |  | 4.00 (-2.40; 10.4) |
|  | *FEV1 reversibility* | | *FEF25-75 reversibility* | |
|  | Model 0 | Model 1^b^ | Model 0 | Model 1^b^ |
| *miR-21-5p* | 8.00x10^-3^ (-0.20; 0.22) | 0.06 (-0.14; 0.26) | 0.08 (-0.34; 0.50) | 0.23 (-0.19; 0.65) |
| *miR-126-3p* | 0.40 (-0.67; 1.47) | -0.31 (-1.36; 0.74) | **3.16 (1.10; 5.23)** | **2.55 (0.35; 4.75)** |
| *miR-133a-3p* | 1.15 (-1.58; 3.88) | -0.73 (-3.40; 1.94) | 1.83 (-3.75; 4.41) | -0.25 (-6.06; 5.56) |
| *miR-145-5p* | 0.32 (-0.38; 1.01) | 7.00x10^-3^ (-0.64; 0.66) | 0.30 (-1.1; 1.71) | -0.15 (1.56; 1.26) |
| *miR-146a-5p* | 2.29 (-3.46; 8.04) | -0.09 (-5.50; 5.32) | **14.7 (3.20; 26.0)** | **11.2 (0.19; 22.7)** |
| *miR-155-5p* | 6.00x10^-3^ (-12.5; 12.5) | 3.85 (-7.53; 15.2) | 13.8 (-11.5; 39.1) | 18.4 (-6.06; 42.8) |
| *miR-221-3p* | 0.57 (-0.68; 1.82) | 0.18 (-1.00; 1.35) | -0.51 (-3.07; 2.05) | -1.09 (-3.63; 1.46) |
| *miR-328-3p* | -0.25 (-1.01; 0.51) | -0.53 (-1.23; 0.17) | 0.07 (-1.47; 1.62) | -0.19 (-1.73; 1.35) |
| *miR-423-3p* | 0.57 (-2.42; 3.55) | 0.13 (-2.74; 2.99) | 7.11 (1.24; 13.0) | 7.26 (1.29; 13.2) |
| Cluster 1 | 0.64 (-1.07; 2.36) | -0.41 (-2.06; 1.25) | 2.43 (-1.04; 5.89) | 1.27 (-2.33; 4.87) |
| Cluster 2 | 0.17 (-1.55; 1.88) | -0.05 (1-65; 1.56) | **4.20 (0.84; 7.57)** | **3.93 (0.58; 7.28)** |
|  | *Breathing difficulties* | | *Irritative cough* | |
|  | Model 0 | Model 1^b^ | Model 0 | Model 1^b^ |
| *miR-21-5p* | -6.00x10^-3^ (-0.02; 5x10^-3^) | -4.00x10^-3^ (-0.01; 6.00x10^-3^) | -5.00x10^-3^ (-0.02; 7.00x10^-3^) | -5.00x10^-3^ (-0-02; 8.00x10^-3^) |
| *miR-126-3p* | -0.01 (-0.09; 0.06) | -0.04 (-0.11; 0.30) | -0.04 (-0.13; 0.04) | -0.05 (-0.14; 0.05) |
| *miR-133a-3p* | -0.06 (-0.24; 0.12) | -0.08 (-0.25; 0.09) | 0.02 (-0.19; 0.23) | -8.00x10^-3^ (-0.23; 0.22) |
| *miR-145-5p* | -0.01 (-0.05; 0.03) | -0.02 (-0.05; 0.02) | -0.02 (-0.06; 0.03) | -0.02 (-0.07; 0.03) |
| *miR-146a-5p* | 0.09 (-0.22; 0.39) | 0.02 (-0.26; 0.29) | -0.04 (-0.40; 0.31) | -0.05 (-0.42; 0.32) |
| *miR-155-5p* | -0.36 (-0.98; 0.27) | -0.13 (-0.69; 0.43) | -0.22 (-0.96; 0.51) | -0.09 (-0.84; 0.66) |
| *miR-221-3p* | -0.04 (-0.12; 0.04) | -0.04 (-0.11; 0.03) | -0.06 (-0.15; 0.02) | -0.07 (-0.16; 0.02) |
| *miR-328-3p* | -0.03 (-0.08; 0.02) | -0.02 (-0.7; 0.03) | 7.00x10^-3^ (-0.05; 0.07) | 0.02 (-0.04; 0.08) |
| *miR-423-3p* | -0.06 (-0.21; 0.10) | -0.07 (-0.21; 0.08) | -0.09 (-0.29; 0.10) | -0.08 (-0.28; 0.13) |
| Cluster 1 | -0.06 (-0.17; 0.05) | -0.07 (-0.17; 0.03) | -0.06 (-0.19; 0.07) | -0.06 (-0.20; 0.08) |
| Cluster 2 | 0.02 (-0.07; 0.11) | 5.00x10^-3^ (-0.08; 0.09) | -0.01 (-0.12; 0.10) | -6.00x10^-3^ (-0.12; 0.11) |

Cluster 1: typified by miR-126-3p, miR-133a-3, miR-145-5p, miR-221-3p and miR-328-3p

Cluster 2: typified by miR-146a-5p and miR-423-3p

^a^: adjusted for: age, sex, atopy, body mass categories according to CDC and asthma defined based on positive bronchodilation or self-reported medical diagnosis with reported symptoms in the previous year and anti-asthma medication

^b^: adjusted for: age, sex, exhaled NO, atopy, body mass categories according to CDC and asthma defined based on positive bronchodilation or self-reported medical diagnosis with reported symptoms in the previous year

FEV1: forced expiratory volume in the first second;

FEF25-75: forced expiratory flow middle portion of FVC

FEV1 reversibility: forced expiratory volume in the first second after bronchodilation;

FEF25-75 reversibility: forced expiratory flow middle portion of FVC after bronchodilation

Significant differences in bold
