# Supplementary material for: Development and validation of exhaled breath condensate microRNAs to identify and endotype asthma in children
Source: PLoS One. 2019 Nov 8;14(11):e0224983. doi: 10.1371/journal.pone.0224983 (PMC6839869; doi:10.1371/journal.pone.0224983)
Supplement: S4 Table — (DOCX) [file pone.0224983.s005.docx]

S4 Table. Associations between miRNAs, exhaled NO, lung function and current symptoms in validation set

|  | *exhaled NO* | | *FVC (% predicted)* | |
| --- | --- | --- | --- | --- |
|  | Model 0 | Model 1^a^ | Model 0 | Model 1^b^ |
|  | β (95% CI) | β (95% CI) | β (95% CI) | β (95% CI) |
| *miR-21-5p* | -3.00x10^-3^ (-0.02; 0.01) | -4.00x10^-3^ (-0.05; 0.04) | -0.31 (-1.08; 0.46) | -1.21 (-3.27; 0.85) |
| *miR-126-3p* | -0.05 (-0.11; 0.02) | -0.06 (-0.18; 0.06) | -2.90 (-6.30; 0.50) | -4.61 (-10.6; 1.38) |
| *miR-133a-3p* | -0.01 (-0.11; 0.08) | -0.06 (-0.28; 0.16) | -2.20 (-7.23; 2.84) | -4.35 (-15.5; 6.83) |
| *miR-145-5p* | -0.02 (-0.05; 0.02) | 2.00x10^-3^ (-0.07; 0.07) | **1.68 (0.09; 3.26)** | 2.04 (-1.36; 5.45) |
| *miR-146a-5p* | -0.27 (-1.17; 0.63) | -1.24 (-3.46; 0.99) | 0.08 (-44.7; 44.8) | 6.16 (-114; 126) |
| *miR-155-5p* | -0.10 (-0.34; 0.15) | -0.10 (-0.72; 0.52) | -8.85 (-21.1; 3.36) | -21.3 (-51.5; 8.86) |
| *miR-221-3p* | -0.05 (-0.19; 0.09) | -0.04 (-0.29; 0.20) | 4.34 (-2.45; 11.2) | 5.10 (-7.44; 17.6) |
| *miR-328-3p* | -0.03 (-0.08; 0.02) | -0.02 (-0.14; 0.10) | 0.28 (-2.25; 2.80) | -1.56 (-7.75; 4.63) |
| *miR-423-3p* | 0.06 (-0.31; 0.43) | 0.04 (-0.63; 0.71) | 10.8 (-8.21; 29.0) | 10.8 (-23.1; 44.6) |
| Cluster 1 | 0.06 (-0.12; 0.01) | -0.05 (-0.18; 0.08) | -2.99 (-6.30; 0.33) | -4.71 (-11.0; 1.57) |
| Cluster 2 | -0.01 (-0.08; 0.06) | -0.05 (-0.22; 0.13) | 0.72 (-2.75; 4.20) | 1.37 (-7.54; 10.3) |
|  | *FEV1 (% predicted)* | | *FEF25-75 (% predicted)* | |
|  | Model 0 | Model 1^b^ | Model 0 | Model 1^b^ |
| *miR-21-5p* | -0.30 (-1.04; 0.43) | -0.67 (-2.66; 1.32) | -0.41 (-1.72; 0.90) | -0.05 (-3.57; 3.46) |
| *miR-126-3p* | -0.67 (-3.98; 2.63) | -2.00 (-7.94; 3.92) | 4.75 (-1.02; 10.5) | 3.99 (-6.30; 14.3) |
| *miR-133a-3p* | -1.77 (-6.59; 3.05) | -2.65 (-13.3; 8.02) | 0.84 (-7.75; 9.42) | 2.99 (-15.7; 21.6) |
| *miR-145-5p* | -0.95 (-2.50; 0.59) | -1.57 (-4.83; 1.69) | 0.96 (-1.80; 3.72) | -1.65 (-7.41; 4.12) |
| *miR-146a-5p* | 6.30 (-36.4; 49.0) | 6.91 (-35.0; 48.8) | 25.5 (-50.1; 101) | 6.74 (-62.4; 75.8) |
| *miR-155-5p* | -7.86 (-19.5; 3.82) | -19.3 (-47.9; 9.39) | -8.28 (-29.2; 12.6) | 1.69 (-19.3; 22.7) |
| *miR-221-3p* | 5.42 (-1.02; 11.9) | 5.23 (-6.59; 17.0) | 5.97 (-5.60; 17.5) | -0.37 (-10.6; 9.88) |
| *miR-328-3p* | 0.12 (-2.30; 2.53) | -0.40 (-6.28; 5.49) | -1.58 (-5.85; 2.69) | -0.40 (-6.28; 5.49) |
| *miR-423-3p* | 10.3 (-7.84; 28.5) | 11.9 (-19.9; 43.7) | 7.64 (-24.9; 40.2) | 12.0 (-44.0; 67.9) |
| Cluster 1 | -1.30 (-4.52; 1.92) | -2.61 (-8.77; 3.54) | 2.41 (-3.31; 8.12) | -0.47 (-10.4; 11.4) |
| Cluster 2 | 1.23 (-2.08; 4.54) | 4.02 (-4.21; 12.3) | 2.18 (-3.71; 8.05) | 9.46 (-4.59; 23.5) |
|  | *FEV1 reversibility* | | *FEF25-75 reversibility* | |
|  | Model 0 | Model 1^b^ | Model 0 | Model 1^b^ |
| *miR-21-5p* | 0.14 (-0.27; 056) | 0.03 (-0.81; 0.86) | 0.24 (-0.68; 1.16) | -0.23 (-1.10; 0.63) |
| *miR-126-3p* | 0.17 (-1.76; 2.10) | -1.15 (-3.59; 1.29) | 2.51 (-1.77; 6.80) | 1.59 (-2.21; 5.38) |
| *miR-133a-3p* | -0.24 (-2.90; 2.42) | 3.13 (-1.10; 7.35) | -0.62 (-6.56; 5.32) | 0.21 (-5.17; 5.59) |
| *miR-145-5p* | 0.25 (-0.65; 1.16) | -0.12 (-1.51; 1.26) | 1.16 (-0.85; 3.18) | 0.47 (-1.38; 2.32) |
| *miR-146a-5p* | 9.58 (-15.1; 34.3) | 7.93 (-17.8; 33.6) | 25.8 (-29.4; 81.0) | 14.1 (-34.3; 62.4) |
| *miR-155-5p* | 0.45 (-6.25; 7.15) | 6.35 (-5.77; 18.5) | 1.87 (-13.1; 16.9) | 3.12 (-10.1; 16.3) |
| *miR-221-3p* | 0.62 (-3.22; 4.45) | 0.03 (-4.98; 5.04) | 4.58 (-3.95; 13.1) | 3.84 (-3.69; 11.4) |
| *miR-328-3p* | -0.91 (-2.30; 0.48) | -0.28 (-2.72; 2.17) | -2.10 (-5.20; 1.00) | -1.10 (-3.94; 1.75) |
| *miR-423-3p* | 1.43 (-9.27; 12.1) | -0.93 (-14.3; 12.5) | 4.02 (-19.9; 27.9) | -2.03 (-22.4; 18.3) |
| Cluster 1 | 0.04 (-1.85; 1.92) | -0.03 (-2.63; 2.58) | 1.74 (-2.44; 5.92) | 1.19 (-2.51; 4.90) |
| Cluster 2 | 0.89 (-1.01; 2.78) | -0.13 (-3.64; 3.37) | 2.59 (-1.65; 6.82) | 0.53 (-3.21; 4.26) |
|  | *Breathing difficulties* | | *Irritative cough* | |
|  | Model 0 | Model 1^b^ | Model 0 | Model 1^b^ |
| *miR-21-5p* | -8.00x10^-3^ (-0.03; 9.00x10^-3^) | -4.00x10^-3^ (-0.02; 0.01) | -2.00x10^-3^ (-0.02; 0.02) | 2.00x10^-3^ (-0.02; 0.02) |
| *miR-126-3p* | -0.05 (-0.16; 0.05) | -0.03 (-0.13; 0.06) | -0.03 (-0.13; 0.07) | -8.00x10^-3^ (-0.11; 0.09) |
| *miR-133a-3p* | -0.03 (-0.15; 0.08) | -0.01 (-0.12; 0.10) | -0.12 (-0.26; 0.02) | -0.11 (-0.24; 0.03) |
| *miR-145-5p* | -0.03 (-0.07; 5.00x10^-3^) | -0.02 (-0.06; 0.01) | -0.04 (-0.09; 6.00x10^-3^) | -0.03 (-0.07; 0.02) |
| *miR-146a-5p* | -0.11 (-1.25; 1.02) | 0.35 (-0.70; 1.40) | 0.61 (-0.72; 1.94) | 1.16 (-0.06; 2.39) |
| *miR-155-5p* | -0.17 (-0.47; 0.14) | -0.14 (-0.41; 0.13) | -0.34 (-0.71; 0.02) | **-****0.37 (-0.70; -0.04)** |
| *miR-221-3p* | -0.07 (-0.24; 0.10) | -0.12 (-0.27; 0.04) | -0.05 (-0.25; 0.15) | -0.08 (-0.27; 0.11) |
| *miR-328-3p* | -0.04 (-0.10; 0.02) | -0.4 (-0.10; 0.02) | 0.04 (-0.04; 0.11) | 0.04 (-0.04; 0.11) |
| *miR-423-3p* | 0.06 (-0.41; 0.54) | -0.02 (-0.45; 0.41) | 0.02 (-0.53; 0.57) | 0.08 (-0.42; 0.59) |
| Cluster 1 | **-0.08 (-016; -2.00x10^-3^)** | -0.07 (-0.14; 9.00x10^-3^) | -0.09 (-0.19; 0.01) | -0.07 (-0.16; 0.03) |
| Cluster 2 | -0.01 (-0.10; 0.07) | -3.00x10^-3^ (-0.08; 0.07) | 0.03 (-0.08; 0.13) | 0.05 (-0.04; 0.15) |

Cluster 1: typified by miR-126-3p, miR-133a-3, miR-145-5p, miR-221-3p and miR-328-3p

Cluster 2: typified by miR-21-5p, miR-146a-5p and miR-423-3p

^a^: adjusted for: age, sex, atopy, body mass categories according to CDC and asthma defined based on positive bronchodilation or self-reported medical diagnosis with reported symptoms in the previous year and anti-asthma medication

^b^: adjusted for: age, sex, exhaled NO, atopy, body mass categories according to CDC and asthma defined based on positive bronchodilation or self-reported medical diagnosis with reported symptoms in the previous year

FEV1: forced expiratory volume in the first second;

FEF25-75: forced expiratory flow middle portion of FVC

FEV1 reversibility: forced expiratory volume in the first second after bronchodilation;

FEF25-75 reversibility: forced expiratory flow middle portion of FVC after bronchodilation

Significant differences in bold
